# Computing *R*_0_ of dynamic models by a definition-based method

**DOI:** 10.1016/j.idm.2022.05.004

**Published:** 2022-05-24

**Authors:** Xiaohao Guo, Yichao Guo, Zeyu Zhao, Shiting Yang, Yanhua Su, Benhua Zhao, Tianmu Chen

**Affiliations:** a State Key Laboratory of Molecular Vaccinology and Molecular Diagnostics, School of Public Health, Xiamen University, Xiamen City, 361102, Fujian Province, People's Republic of China; b Université de Montpellier, CIRAD, Intertryp, IES, Université de Montpellier-CNRS, Montpellier, France

**Keywords:** Definition-based method, Dynamics model, Basic reproduction number, Next-generation method

## Abstract

**Objectives:**

Computing the basic reproduction number (*R*_0_) in deterministic dynamical models is a hot topic and is frequently demanded by researchers in public health. The next-generation methods (NGM) are widely used for such computation, however, the results of NGM are usually not to be the true *R*_0_ but only a threshold quantity with little interpretation. In this paper, a definition-based method (DBM) is proposed to solve such a problem.

**Methods:**

Start with the definition of *R*_0_, consider different states that one infected individual may develop into, and take expectations. A comparison with NGM has proceeded. Numerical verification is performed using parameters fitted by data of COVID-19 in Hunan Province.

**Results:**

DBM and NGM give identical expressions for single-host models with single-group and interactive *R*_*ij*_ of single-host models with multi-groups, while difference arises for models partitioned into subgroups. Numerical verification showed the consistencies and differences between DBM and NGM, which supports the conclusion that *R*_0_ derived by DBM with true epidemiological interpretations are better.

**Conclusions:**

DBM is more suitable for single-host models, especially for models partitioned into subgroups. However, for multi-host dynamic models where the true *R*_0_ is failed to define, we may turn to the NGM for the threshold *R*_0_.

## 1 Introduction

The basic reproduction number (*R*_0_) is ‘one of the foremost and most valuable ideas that mathematical thinking has brought to epidemic theory’ (Heesterbeek & Dietz, 1996). It is an epidemiology metric used to quantify the transmissibility of infectious diseases. *R*_0_ is defined as the expectation of secondary cases produced by one infected individual in the entirely susceptible population, during its lifespan as infectious. The definition assumes that in the absence of any intervention (Becker et al., 2006), like quarantine, vaccination, etc., that is, all individuals are not immune. In its definition, *R*_0_ is related to epidemiology characteristics, including the infectious period of a person who becomes infected, the frequency of contact during the infectious period, and the probability of transmission per contact between a susceptible individual and an infected person. Accordingly, the effectiveness of contact tracing and case isolation in controlling coronavirus disease 2019 (COVID-19) depends partly on the precise estimation of *R*_0_, with *R*_0_ of 1.5, outbreaks were controllable if 50% of contacts were traced. And with *R*_0_ of 2.5 and 3.5, more than 70% and 90% of contacts had to be traced, respectively (Hellewell et al., 2020).

*R*_0_ has the threshold property that if *R*_0_ < 1, each infected individual produces, on average, less than one secondary case during its lifespan as infectious, and therefore the epidemic will gradually disappear, whereas if *R*_0_ > 1, the disease may lead to a large number of new infections. Estimation of the correct *R*_0_ is crucial in an epidemic emergency response because it informs later direction for control measures and can be compared to the effective reproduction number after interventions (Ma et al., 2022). Besides, it is also used to estimate the vaccine coverage needed for herd immunity (Garnett, 2005), the coverage should be greater than 1-1/*R*_0_ (Desenclos, 2011; Fine et al., 2011), considering the effectiveness of vaccines is not 100%. And as transmissibility evolves, herd immunity thresholds can be estimated by modified *R*_0_ (Gomes et al., 2022; Montalbán et al., 2020). Furthermore, following the results of the basic reproduction number, optimal control analyses were developed to reduce disease transmission, including co-infection (Tchoumi et al., 2021), as well as the most cost-efficient measures (Asamoah et al., 2022).

Since the concept of basic reproduction number was proposed by Böckh in 1886 till today (which George Macdonald applied it in modern epidemiology for the first time in 1952) (Heesterbeek et al., 2015; Heesterbeek, 2002; Heffernan et al., 2005), there are mainly two kinds of methods to calculate *R*_0_: driven by data and through dynamic models. Data-driven approaches, including intrinsic growth rate (Chowell et al., 2004), the final size equation (Arino et al., 2007), the average age of infection (Ajelli et al., 2008), etc. Through dynamic models, the methods separate into three general categories: Jacobian approach, the next-generation method (Martcheva, 2015), and estimate directly by *R*_0_'s definition (Batabyal, 2021; Dearlove & Wilson, 2013; Getz et al., 2018; Hethcote, 2000). The next-generation method is likely the most frequently used method to calculate *R*_0_ due to its generality, as *R*_0_ is always computable if the compartment model is given. However, the definition, calculation, and interpretation of *R*_0_ derive from NGM are anything but simple. Thoroughly grasping the mathematical method of *R*_0_ calculation is still difficult for most practitioners and researchers in public health. Estimate *R*_0_ directly through definition has been described in basic dynamic models without an explicit step-by-step derivation process (Batabyal, 2021; Dearlove & Wilson, 2013; Getz et al., 2018; Hethcote, 2000). Moreover, few of these methods agree with each other, even when based on the same dataset (Li & Blakeley, 2011). Therefore, this study formularizes and generalizes an intelligible and straightforward definition-based method (DBM) for computing *R*_0_ of dynamic models of single host species for the first time, from which one can work out the expression of *R*_0_ with just pen and paper. The result that this method produces is mutually coherent with the next-generation method (NGM) and is typically equivalent to the Van den Driessche and Watmough approach. Further, the result differs in computing *R*_0_ for an entire population with multi-group models, while DBM gives the true epidemiological interpretation.

## 2 Materials and methods

### 2.1 Modeling

We divide the compartmental dynamic models into four classes and associate each class with some applied models as examples. The assumptions, compartment definition, and parameter estimation to those models can be found in corresponding references. The flowchart, ordinary differential equations, and interpretations to variables and parameters are included in the supplementary materials. All parameters in compartmental models, except the transmission rate coefficient, which is usually obtained by data fitting, are obtained either from references or estimated using statistical inferences on original data.

### 2.2 Models of single host population with single transmission route (SS)

This part considers only one host species and one route of transmission, as the widely used Susceptible-Infectious-Recovered (SIR) (Cooper et al., 2020), Susceptible-Exposed-Infectious-Recovered (SEIR) (Ansumali et al., 2020), and Susceptible-Exposed-Infectious/Asymptomatic-Recovered (SEIAR) (Chen et al., 2020) models.

### 2.3 Models of single host population divided by subgroups with single transmission route (SDS)

**Fig. 1:**
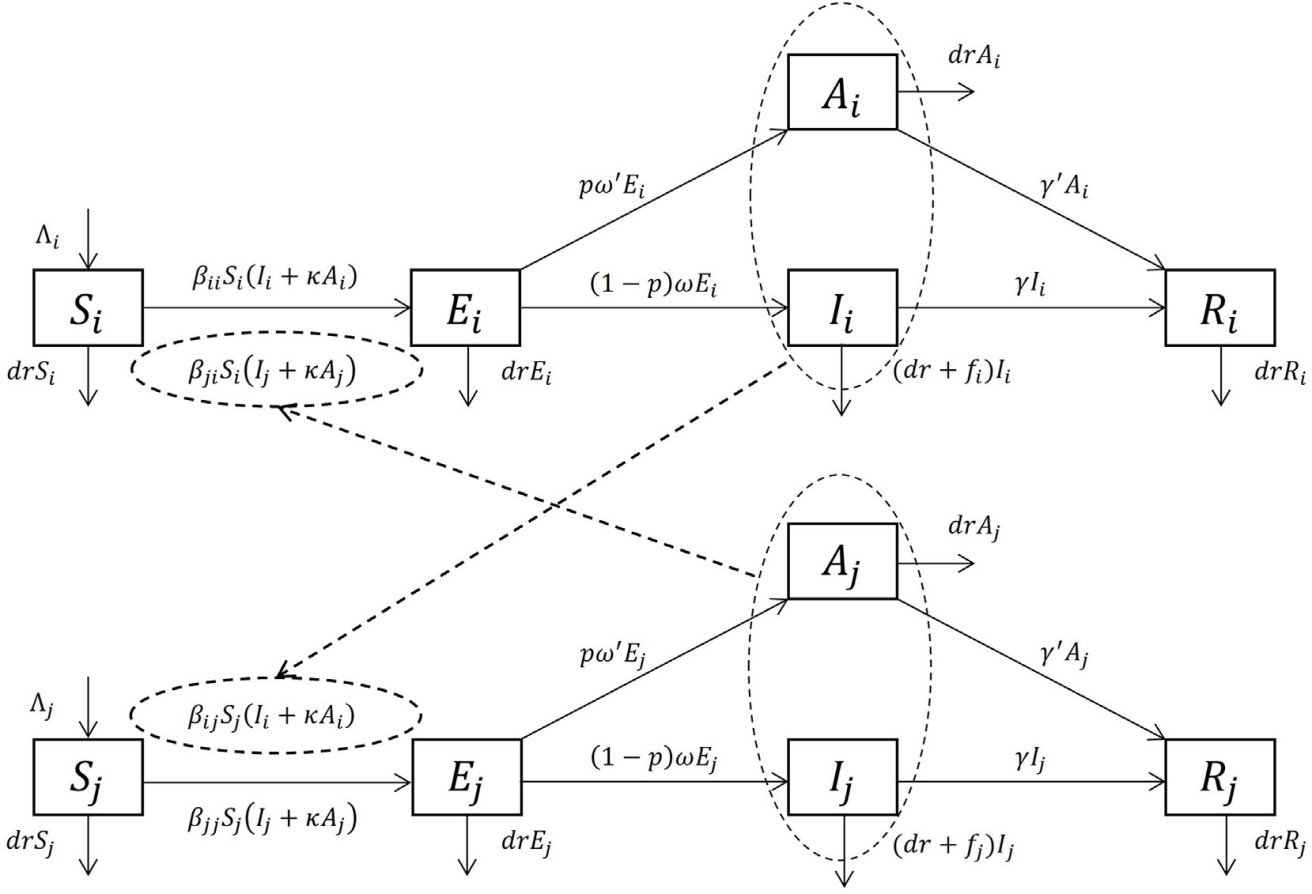
Flowchart of Multi Groups SEIAR model. The subscript i denotes the variable or parameter is specified to the i-th group. n denotes the total number of groups. Variables $S_{i},E_{i},I_{i},A_{i},R_{i},i=1,2,\cdots ,n$ represent the susceptible, exposed, symptomatic infectious, asymptomatic infectious and recovered population in group i; $N_{i}=S_{i}+E_{i}+I_{i}+A_{i}+R_{i}$ is the population size of group $i;N=N_{1}+N_{2}+\cdots +N_{n}$ is the total population size. Parameters $\Lambda_{i}$ are constant birth coefficients (for age-grouped model, only one $\Lambda_{i}$ of the youngest age group is nonzero); $dr_{i}$ denotes the mortality rate of group $i;\beta_{ij}$ is the coefficients describing the daily transmission rate from group i to group j;κ is the relatively of transmission ability of asymptomatic cases compared with the symptomatic cases, it is assumed to be group irrelevant; $f_{i}$ is the case fatality rate of group i (if group irrelevant, then f); $p_{i}$ is the probability that one infected individual in group i will developed into a asymptomatic case (if group irrelevant, then $p);\omega_{i}$ is inverse of the average latent period of the symptomatic population in the i-th group, it is used to quantify the remove rate of compartment $E_{i}$ (the inverse of incubation period is usually adopted for practice); $\omega_{i}^{\text{′}}$ is the inverse of the average latent period of the asymptomatic population in the i-th group; $\gamma_{i}$ is the inverse of average infectious period for symptomatic cases; $\gamma_{i}^{\text{′}}$ is the inverse of average infectious period for asymptomatic. The solid arrows represent the transitions between variables.

The multi-group SEIAR model of the previous studies (Fig. 1) (Lin et al., 2021) is considered. For the universality and generality, we consider a modified model with a nonzero birth rate and death rate and assume the transition rate of A and I are nonequal.

### 2.4 Models of single host population with multi transmission routes (SM)

**Fig. 2:**
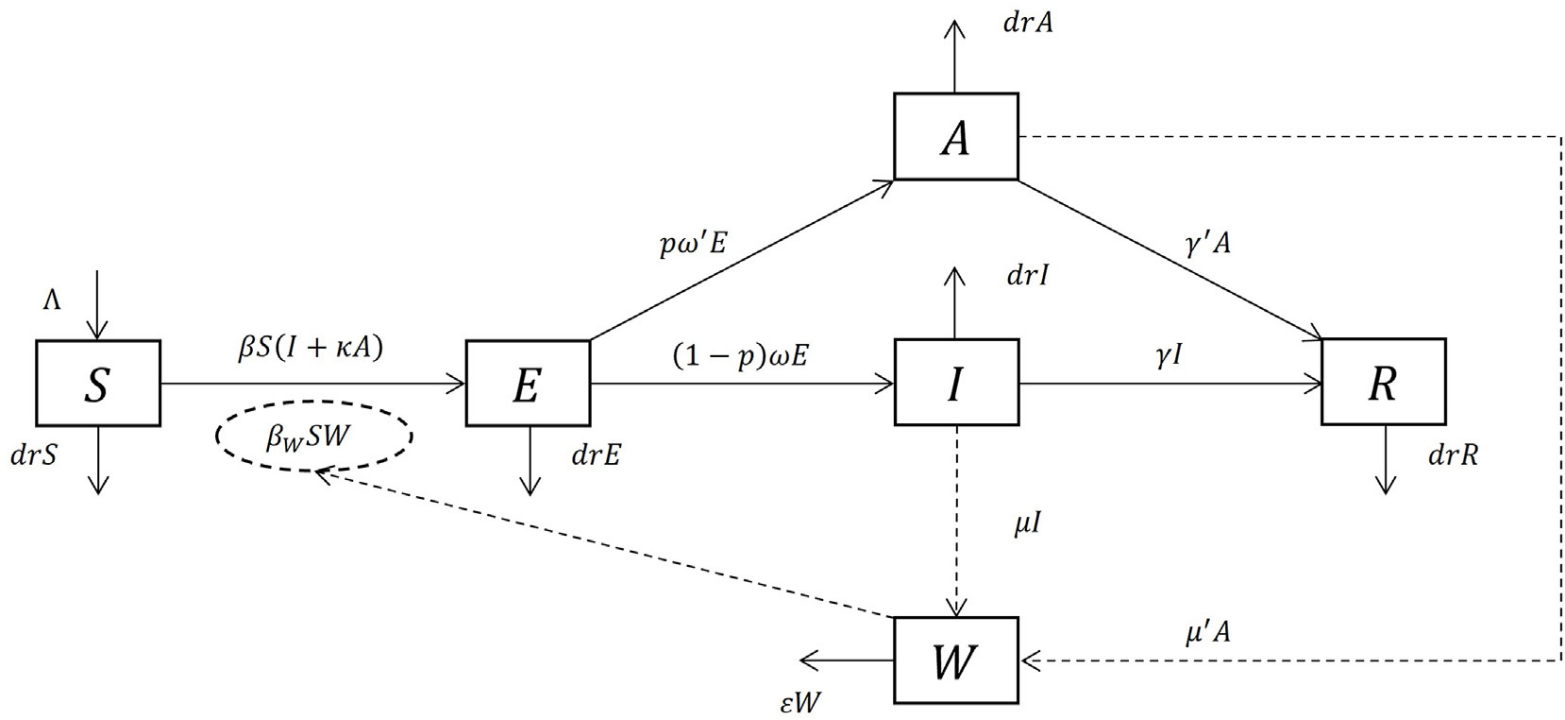
Flowchart of SEIARW model. Variables S,E,I,A,R, W represent population of susceptible, exposed, symptomatic infectious, asymptomatic infectious, recovered and pathogen in reservoir. Parameter Λ is a constant birth coefficient; β is the human-to-human transmission rate coefficient; $\beta_{W}$ is the reservoir-to-human transmission rate coefficient; κ is the relatively of transmission ability of asymptomatic cases compared with the symptomatic cases; $d_{r}$ is the natural death rate; f is the fatality; p represent the proportion of progressing to asymptomatic infectious stage; ω is the inverse of the average latent period of the symptomatic cases; $\omega^{\text{′}}$ is the inverse of the average latent period of the asymptomatic cases; γ is the inverse of the infectious period of symptomatic cases; $\gamma^{\text{′}}$ is the inverse of average infectious period of asymptomatic cases. The solid arrows represent the transitions between variables; the dashed arrows represent pathogen shedding into environment.

For single host models, there may be two or more routes of transmission. As the widely used Susceptible-Exposed-Infectious/Asymptomatic-Recovered-Water (SEIARW) model (Fig. 2) (Chen et al., 2014, Chen et al., 2014), there are two routes of transmission: person to person and environment (water and food) to person.

### 2.5 Models of multi-host (MH)

The multi-host models involve two or more host populations of different species, like models for dengue fever (Yi et al., 2019), brucellosis (Sun et al., 2020), and severe fever with thrombocytopenia syndrome (Zhang et al., 2021), in which the transmission happened inside and between those different species. The vector-borne disease is a special case of multi-host models, that infections will never occur inside each one of host species, and can be formularized by setting the transmission coefficients inside each group be zero in the multi-host models.

### 2.6 Study design

**Fig. 3:**
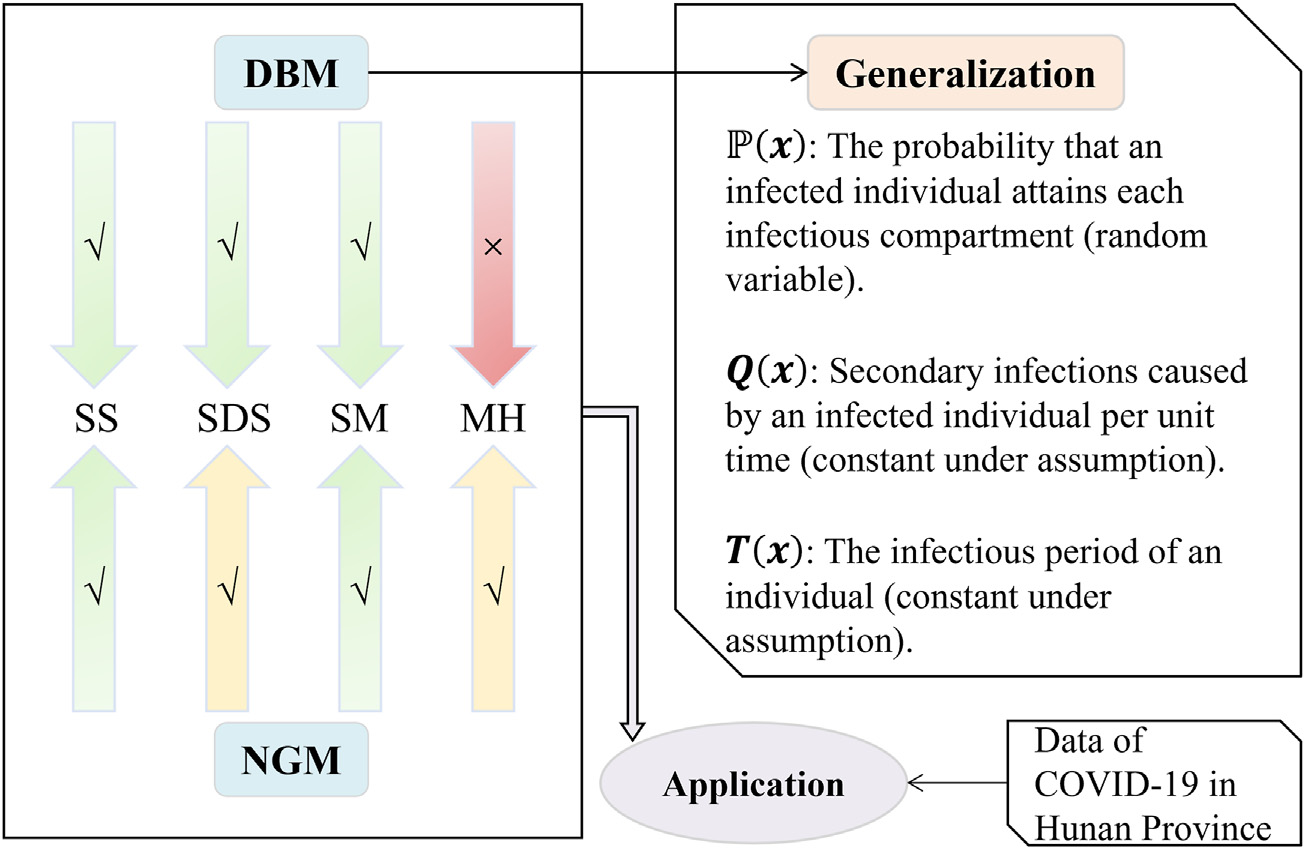
Flowchart of study design. SS, SDS, SM, MH are models of four categories. Green checkmark arrows denote the method that works well for those models; yellow checkmark arrows denote the method is computable while it gives little epidemiological interpretation; the red cross mark arrow denotes the method is inadequate for those models.

We proposed generalized procedures of a definition-based method (DBM) for computing *R*_*0*_ and compared it with the frequently used next-generation method (NGM) on models of SS, SDS, SM, MH. Numerical validation for SDS is proceeded using data of COVID-19 in Hunan Province. (Fig. 3).

### 2.7 Computation of *R*_0_

#### 2.7.1 Definition-Based method (DBM)

**Fig. 4:**
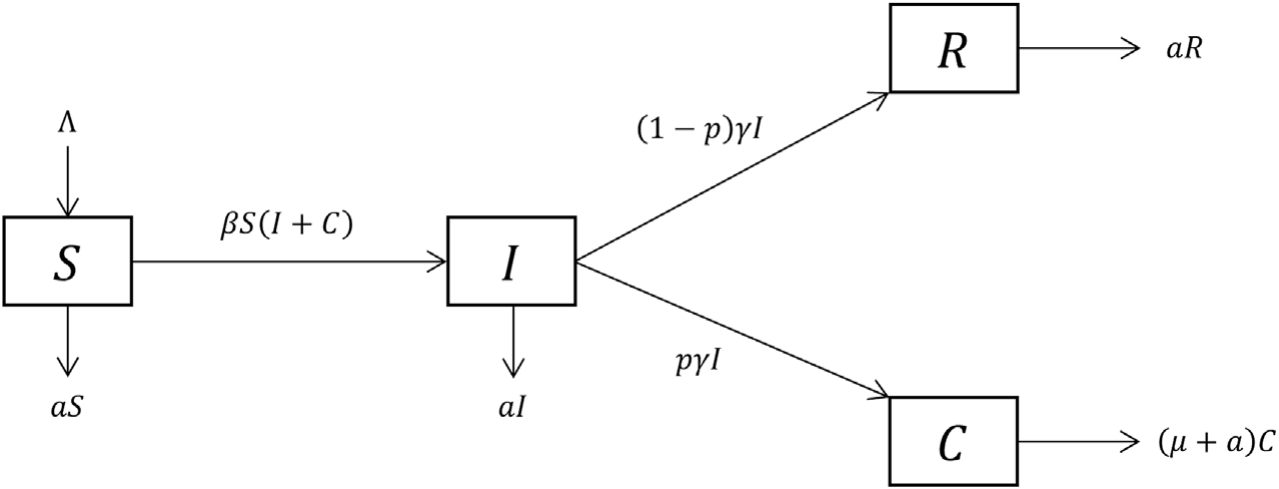
Flowchart of SIRC model. Variables S, I, R, C represent population of susceptible, acute infected, recovered, and chronic infected. Parameters Λ represent birth rate; β is the transmission rate coefficient; a is the natural death rate; μ is the fatality; p represent the proportion of progressing to chronic stage; γ is the inverse of the duration of mean acute infection. The solid arrows represent the transitions between variables.

For an easier understanding of the proposed method, the procedure of a specific example of Susceptible-Acute infectious-Recovered/Chronic infectious (SIRC) model (Cui et al., 2020) (Fig. 4) will be proposed after each generalized step is stated.

Step 1 For any infected individual *x*, we start from the initial compartment of *x* [usually exposed (*E*) or infectious (*I*)] and compute the probability P(x) that *x* attains each infectious compartment. For the SIRC model, we have:

$$\begin{array}{cc} & P\left(x\in I\right)=1\\ & P\left(x\in C|x\in I\right)=\frac{p\gamma }{a+p\gamma +\left(1-p\right)\gamma }\\ & P\left(x\in C\right)=P\left(x\in I\right)P\left(x\in C|x\in I\right)=\frac{p\gamma }{a+p\gamma +\left(1-p\right)\gamma } \end{array}$$

Step 2 For all infectious compartments $[U_{1},U_{2},\ldots ,U_{r}$] that *x* might attain, compute the newly infection rate for the case that *x* in $U_{i},\;i=1,2,\ldots ,r,$ by letting $U_{i}$ equals 1, and other $U_{j},j\neq i$ equals zero in the term of newly infection ($\text{e}.\text{g}.,\beta Sf\left(U_{1},U_{2},\ldots ,U_{r}\right)$, where f is a multi-variable function). For the SIRC model, by letting (*I*, *C*) = (1, 0) and (0, 1) respectively, one obtains the secondary infection produced by *x* per unit time, for two cases that *x* in acute infectious (*I*) and *x* in chronic infectious (*C*):

Q(x∈I)=Q(x∈C)=βS

Step 3 Compute the length of time interval T(x) that x will stay in each infectious compartment using compartment removing rate (fatality of the disease, recovery rate, and natural death rate, usually set to be constants). For the SIRC model:

$$T\left(x\right)=\left\{ \begin{array}{cc} 1/\left(a+p\gamma +\left(1-p\right)\gamma \right), & \;if\;x\in I\\ 1/\left(\mu +a\right), & \;if\;x\in C \end{array}\right.$$

Step 4 Assuming that the disease is currently at an initial stage of development, the number of infected takes only a small portion of the total population. This assumption implies Q(x) can be treated as constant during the time interval T(x).

Step 5 Taking expectations to eliminate the stochasticity of different infectious compartments *x* might develop into. The effective reproduction number is obtained:

$$\begin{array}{l} R_{eff}=\underset{all\;infectious\;State_{i}}{\sum }P\left(x\in State_{i}\right)Q\left(x\in State_{i}\right)T\left(x\in State_{i}\right) \end{array}$$

For the SIRC model:

$$\begin{array}{ll} R_{eff} & =P\left(x\in I\right)Q\left(x\in I\right)T\left(x\in I\right)+P\left(x\in C\right)Q\left(x\in C\right)T\left(x\in C\right)\\ & =1\times \beta S\times \frac{1}{a+p\gamma +\left(1-p\right)\gamma }+\frac{p\gamma }{a+p\gamma +\left(1-p\right)\gamma }\times \beta S\times \frac{1}{\mu +a}\\ & =\frac{\beta S}{a+\gamma }+\frac{p\gamma \beta S}{\left(\mu +a\right)\left(a+p\right)} \end{array}$$

Step 6 The *R*_0_ is obtained by assuming the entire population is susceptible and letting S=N in the expression of $R_{eff}$. For the SIRC model:

$$\begin{array}{c} R_{0}=\frac{\beta N}{a+\gamma }+\frac{p\gamma \beta N}{\left(\mu +a\right)\left(a+p\right)} \end{array}$$

Note that in Step 1, if the flowchart is bidirectional, then the computation of reachable probability may involve the assumption of Markov property and the use of the transition matrix.

#### 2.7.2 Next-generation method (NGM)

The most frequently used Van den Driessche and Watmough approach in NGM is chosen as candidate method for comparison. The steps of NGM are listed as follows:

Step 1 Divide all compartments into two categories: non-infected compartments (including susceptible, recovered, and fully immunized) and infected compartments

Step 2 Write down the corresponding differential equations of the transition graph and divide the derivative vector of infected compartments into two parts: the first F is newly infected rate, and the second V is the rate of transition between different compartments.

Step 3 Take derivatives with respect to infected compartments for vectors F and V, and obtain the Jacobi matrices FandV.

Step 4 Compute the next generation matrix $FV^{-1}$ and its leading eigenvalue $\lambda_{max}\left(FV^{-1}\right)$.

Step 5 Substitute the disease-free equilibrium into $\lambda_{max}\left(FV^{-1}\right)$, and the $R_{0}$ defined by NGM is obtained.

### 2.8 Analysis and validation

The symbolic toolbox of MATLAB (R2021a) was used to perform the inverse and eigenvalue operations for symbolic matrices. Numerical experiments of DBM and NGM were performed using fitted transmission parameters and other parameters in a published age-specific multi-group SEIAR model (Zhao et al., 2020). All codes of computation are available for download on GitHub and provided in supplementary materials (S1 Text: Supplementary Methods and Results).

## 3 Results

### 3.1 R_0_ for SS

For SS model, the results of DBM and NGM are mutually equivalent. Results of some popular models are listed as follows (derivations and detailed model frameworks are provided in S1 Text: Supplementary Methods and Results):

SIR:

(1) $$\begin{array}{l} R_{0}=\frac{\beta N}{\left(\gamma +d_{r}\right)} \end{array}$$

SEIR:

(2) $$\begin{array}{l} R_{0}=\frac{\omega \beta N}{\left(\omega +d_{r}\right)\left(\gamma +d_{r}\right)} \end{array}$$

SEIAR:

(3) $$\begin{array}{l} R_{0}=\frac{\beta N}{p\omega^{\text{′}}+\left(1-p\right)\omega +d_{r}}\left[\frac{\kappa p\omega^{\text{′}}}{\gamma^{\text{′}}+d_{r}}+\frac{\left(1-p\right)\omega }{\gamma +f+d_{r}}\right] \end{array}$$

SIRC:

(4) $$R_{0}=\frac{\beta N}{a+\gamma }+\frac{p\gamma \beta N}{\left(a+\gamma \right)\left(\mu +a\right)}$$

### 3.2 Interactive R_ij_ and R_0_ for SDS

The interactive *R*_*ij*_ between groups in a multi-group model, which is defined as the expectation of secondary infections that one infected individual in group *i* will produce in an entirely susceptible population of group *j* during its lifespan as infectious, is a quantity of great interest. All *R*_*ij*_ forms a matrix that depicts transmission between groups.

We use a multi-group SEIAR model (Zhao et al., 2020) as an example to illustrate the behaviors of DBM and NGM. The flowchart of this model is shown in (Fig. 1). The following derivation of DBM and NGM both leads to an identical result of *R*_*ij*_. And in constructing $R_{0}$ for the entire population via *R*_*ij*_ matrix, we conclude that DBM are more suitable than NGM.

#### 3.2.1 DBM for interactive R_ij_ (SDS)

The derivation steps using DBM are as follows:

Step 1 For any infected individual $x_{i}$ in group i, there are three possible states: $E_{i}$, $I_{i}$, and $A_{i}$. The first step is to consider the initial state $E_{i}$ and compute the probability of $x_{i}$ passing other infectious compartments. It is shown in the transition graph (Fig. 1), that for individual $x_{i}\in E_{i}$, it might develop into $d_{r}E_{i}$ (natural death during exposure), $I_{i}$ and $A_{i}.$ Since $P\left(x_{i}\in E_{i}\right)\;=\;1,$ the probabilities are computed by ratio of transition rate given in the transition graph:

$$\begin{array}{cc} & P\left(x_{i}\in d_{r}E_{i}\right)=P\left(x_{i}\in d_{r}E_{i}|x_{i}\in E_{i}\right)P\left(x_{i}\in E_{i}\right)=\frac{d_{r}}{p\omega^{\text{′}}+\left(1-p\right)\omega +d_{r}}\;\left(dead\right)\\ & P\left(x_{i}\in A_{i}\right)\;=\;P\left(x_{i}\in A_{i}|x_{i}\in E_{i}\right)P\left(x_{i}\in E_{i}\right)=\frac{p\omega^{\text{′}}}{p\omega^{\text{′}}+\left(1-p\right)\omega +d_{r}}\\ & P\left(x_{i}\in I_{i}\right)=\;P\left(x_{i}\in I_{i}|x_{i}\in E_{i}\right)P\left(x_{i}\in E_{i}\right)=\frac{\left(1-p\right)\omega }{p\omega^{\text{′}}+\left(1-p\right)\omega +d_{r}} \end{array}$$

where $P\left(x_{i}\in E_{i}\right)$ denotes the probability that individual $x_{i}$ will develop into the compartment $E_{i}$; and $P\left(x_{i}\in I_{i}|x_{i}\in E_{i}\right)$ denotes the conditional probability of $x_{i}$ will develop into the compartment $I_{i}$ with $x_{i}\in E_{i}$ known.

Step 2 Letting one of three components of $\left(d_{r}E_{i},I_{i},A_{i}\right)$ equals to 1, and others equal 0 respectively and substitute into the term of newly infection rate $\beta_{ij}S_{j}\left(I_{i}+\kappa A_{i}\right)$, one obtains the number of secondary infections in group j, that one infected individual $x_{i}$ will produce per unit time for the different compartments that $x_{i}$ might attain:

$$Q\left(x_{i}\right)=\left\{ \begin{array}{cc} 0, & \;if\;x_{i}\in d_{r}E_{i}\;\left(dead\right)\\ \beta_{ij}\kappa S_{j}, & \;if\;x_{i}\in A_{i}\\ \beta_{ij}S_{j}, & \;if\;x_{i}\in I_{i} \end{array}\right.$$

Step 3 Take compartment $I_{i}$ as an example, if the death rates are omitted ($d_{r}=f=0$), then the infectious period of individual $x_{i}$ is 1/γ (usually, the average course of infection is adopted). For the case that death rates are considered, we combined the terms $\left(d_{r}+f\right)I_{i}\;\text{and}\;\gamma I_{i}$, and hence the infectious period is $1/\left(d_{r}+f+\gamma \right)$. Similar procedures obtain the infectious period for other compartments of $x_{i}$ might attain:

$$T\left(x_{i}\right)=\left\{ \begin{array}{cc} 0, & \;if\;x_{i}\in d_{i}E_{i}\;\left(dead\right)\\ 1/\left(\gamma^{\text{′}}+d_{r}\right), & \;if\;x_{i}\in A_{i}\\ 1/\left(f_{i}+\gamma +d_{r}\right), & \;if\;x_{i}\in I_{i} \end{array}\right.$$

Step 4 At the beginning of disease transmission, S≈N, and S/N is nearly a constant. As a good 1-order approximation, it is natural to assume that $Q\left(x_{i}\right)$ is constant during the time interval $T\left(x_{i}\right)$.

Step 5 By taking expectations, the expectation of secondary infections in group j that one infected individual in group i will produce during its lifespan as infectious is given by:

$$\begin{array}{ll} R_{eff,ij}= & P\left(x_{i}\in d_{r}E_{i}\right)\times 0\times 0\\ & +P\left(x_{i}\in A_{i}\right)\times \beta_{ij}\kappa S_{j}\times \left(1/\left(\gamma^{\text{′}}+d_{r}\right)\right)\\ & +P\left(x_{i}\in I_{i}\right)\times \beta_{ij}S_{j}\times \left(1/\left(f_{i}+\gamma +d_{r}\right)\right) \end{array}$$

Step 6 Assuming group j is entirely susceptible, that by letting $S_{j}=N_{j}$, one obtains the $R_{ij}$ defined in the beginning:

(5) $$\begin{array}{l} R_{ij}=\frac{\beta_{ij}N_{j}}{d_{r}+p\omega^{\text{′}}+\left(1-p\right)\omega }\left(\frac{\kappa p\omega^{\text{′}}}{\left(\gamma^{\text{′}}+d_{r}\right)}+\frac{\left(1-p\right)\omega }{\left(\gamma +d_{r}+f_{i}\right)}\right) \end{array}$$

#### 3.2.2 NGM for interactive R_ij_ (SDS)

The derivation steps using NGM are as follows:

Step 1 Assume that the host population is divided into n groups. Firstly we divide all 5n compartments $\left(S_{i},E_{i},A_{i},I_{i},R_{i}\right)∥,i=1,2,\cdots ,n$ into two categories: the first $\left(E_{i},A_{i},I_{i}\right)∥,i=1,2,\cdots ,n$ are infected compartments, and the second $\left(S_{i},R_{i}\right)∥,i=1,2,\cdots ,n$ are non-infected compartments.

Step 2 Divide the derivation of $\left(E_{i},A_{i},I_{i}\right)∥,i=1,2,\cdots ,n$ into two parts: the first part F denotes the rate of newly infection, and the second part V denotes the transition inside the infected compartments.

$$\begin{array}{ll} \frac{d}{dt}\left[\begin{array}{l} E_{i}\\ A_{i}\\ I_{i} \end{array}\right] & =\left[\begin{array}{l} \overset{n}{\underset{j=1}{\sum }}\beta_{ji}S_{i}\left(I_{j}+\kappa A_{j}\right)-p\omega_{2}E_{i}-\left(1-p\right)\omega_{1}E_{i}-d_{r}E_{i}\\ p\omega_{2}E_{i}-\gamma^{\text{′}}A_{i}-d_{r}A_{i}\\ \left(1-p\right)\omega_{1}E_{i}-\left(f_{i}+\gamma \right)I_{i}-d_{r}I_{i} \end{array}\right]\\ & =\left[\begin{array}{l} \overset{n}{\underset{j=1}{\sum }}\beta_{ji}S_{i}\left(I_{j}+\kappa A_{j}\right)\\ 0\\ 0 \end{array}\right]-\left[\begin{array}{l} p\omega_{2}E_{i}+\left(1-p\right)\omega_{1}E_{i}+d_{r}E_{i}\\ -p\omega_{2}E_{i}+\gamma^{\text{′}}A_{i}+d_{r}A_{i}\\ -\left(1-p\right)\omega_{1}E_{i}+\left(f_{i}+\gamma \right)I_{i}+d_{r}I_{i} \end{array}\right]\\ & :=F_{i}-V_{i} \end{array}$$

Step 3 Take derivatives of 3n-dimension vector-valued function F and V, respect variables $\left(E_{i},A_{i},I_{i}\right),i=1,2,\cdots ,n$.

$$F=\left[\begin{array}{llll} F_{11} & F_{12} & \cdots & F_{1n}\\ F_{21} & F_{22} & \cdots & F_{2n}\\ \vdots & \vdots & \ddots & \vdots \\ F_{n1} & F_{n2} & \cdots & F_{nn} \end{array}\right],\;V=\left[\begin{array}{llll} V_{11} & V_{12} & \cdots & V_{1n}\\ V_{21} & V_{22} & \cdots & V_{2n}\\ \vdots & \vdots & \ddots & \vdots \\ V_{n1} & V_{n2} & \cdots & V_{nn} \end{array}\right]$$

where

$$F_{ij}=\left[\begin{array}{lll} \partial F_{i}\left(1\right)/\partial E_{j} & \partial F_{i}\left(1\right)/\partial A_{j} & \partial F_{i}\left(1\right)/\partial I_{j}\\ \partial F_{i}\left(2\right)/\partial E_{j} & \partial F_{i}\left(2\right)/\partial A_{j} & \partial F_{i}\left(2\right)/\partial I_{j}\\ \partial F_{i}\left(3\right)/\partial E_{j} & \partial F_{i}\left(3\right)/\partial A_{j} & \partial F_{i}\left(3\right)/\partial I_{j} \end{array}\right]=\left[\begin{array}{lll} 0 & S_{i}\;\beta_{ji}\;\kappa & S_{i}\;\beta_{ji}\\ 0 & 0 & 0\\ 0 & 0 & 0 \end{array}\right]$$

$$\begin{array}{l} V_{ij}=\left[\begin{array}{lll} \partial V_{i}\left(1\right)/\partial E_{j} & \partial V_{i}\left(1\right)/\partial A_{j} & \partial V_{i}\left(1\right)/\partial I_{j}\\ \partial V_{i}\left(2\right)/\partial E_{j} & \partial V_{i}\left(2\right)/\partial A_{j} & \partial V_{i}\left(2\right)/\partial I_{j}\\ \partial V_{i}\left(3\right)/\partial E_{j} & \partial V_{i}\left(3\right)/\partial A_{j} & \partial V_{i}\left(3\right)/\partial I_{j} \end{array}\right]\\ \;\;\;\;=\delta_{ij}\left[\begin{array}{lll} d_{r}+\omega^{\text{′}}\;p+\omega \;\left(1-p\right) & 0 & 0\\ -\omega^{\text{′}}\;p & d_{r}+\gamma^{\text{′}} & 0\\ -\omega \;\left(1-p\right) & 0 & d_{r}+f_{i}+\gamma \end{array}\right] \end{array}$$

where $\delta_{ij}$ is the Kronecker Delta, i.e., $\delta_{ij}=1$ for i=j, and $\delta_{ij}=0$ for i≠j.

We call $F_{ij}$ the newly infection matrix of group j to group i, call $V_{ii}$ be the transition matrix of group i.

The inverse of $V_{ii}$ is further computed:

$$V_{ii}^{-1}=\left[\begin{array}{lll} \frac{1}{d_{r}+\omega -\omega \;p+\omega^{\text{′}}\;p} & 0 & 0\\ \frac{\omega^{\text{′}}\;p}{\left(d_{r}+\gamma^{\text{′}}\right)\;\left(d_{r}+\omega -\omega \;p+\omega^{\text{′}}\;p\right)} & \frac{1}{d_{r}+\gamma^{\text{′}}} & 0\\ \frac{\omega \;\left(1-p\right)}{\left(d_{r}+f_{i}+\gamma \right)\;\left(d_{r}+\omega -\omega \;p+\omega^{\text{′}}\;p\right)} & 0 & \frac{1}{d_{r}+f_{i}+\gamma } \end{array}\right]$$

Step 4 Construct the next generation matrix $M=FV^{-1}$

Block matrices M,FandV:

$$M=\left[\begin{array}{llll} M_{11} & M_{12} & \cdots & M_{1n}\\ M_{21} & M_{22} & \cdots & M_{2n}\\ \vdots & \vdots & \ddots & \vdots \\ M_{n1} & M_{n2} & \cdots & M_{nn} \end{array}\right]=\left[\begin{array}{llll} F_{11} & F_{12} & \cdots & F_{1n}\\ F_{21} & F_{22} & \cdots & F_{2n}\\ \vdots & \vdots & \ddots & \vdots \\ F_{n1} & F_{n2} & \cdots & F_{nn} \end{array}\right]\left[\begin{array}{llll} V_{11}^{-1} & & & \\ & V_{22}^{-1} & & \\ & & \ddots & \\ & & & V_{nn}^{-1} \end{array}\right]$$

where

$$\begin{array}{ll} M_{ij} & =\overset{n}{\underset{k=1}{\sum }}F_{ik}V_{kj}^{-1}=F_{ij}V_{jj}^{-1}=\left[\begin{array}{lll} \frac{\beta_{ji}N_{j}}{d_{r}+p\omega^{\text{′}}+\left(1-p\right)\omega }\left(\frac{\kappa p\omega^{\text{′}}}{\left(\gamma^{\text{′}}+d_{r}\right)}+\frac{\left(1-p\right)\omega }{\left(\gamma +d_{r}+f_{j}\right)}\right) & \frac{S_{i}\;\beta_{ji}\;\kappa }{d_{r}+\gamma^{\text{′}}} & \frac{S_{i}\;\beta_{ji}}{d_{r}+f_{j}+\gamma }\\ 0 & 0 & 0\\ 0 & 0 & 0 \end{array}\right] \end{array}$$

Step 5 We use the leading eigenvalue of $M_{ij}$ at the disease-free equilibrium to measure the infectivity of group j to group i:

(6) $$\begin{array}{l} \lambda_{max}\left(M_{ij}\right)=\frac{\beta_{ji}N_{j}}{d_{r}+p\omega^{\text{′}}+\left(1-p\right)\omega }\left(\frac{\kappa p\omega^{\text{′}}}{\left(\gamma^{\text{′}}+d_{r}\right)}+\frac{\left(1-p\right)\omega }{\left(\gamma +d_{r}+f_{j}\right)}\right) \end{array}$$

We can notice that equations (5), (6) are mutually equivalent, that is, NGM is equivalent to DBM for the interactive $R_{ij}$ of this model.

### 3.3 R_0_ for Entire population of SDS

#### 3.3.1 DBM for *R*_0_ (SDS)

Let $R\left(x_{i},j\right)$ denote the random variable describe the secondary infections that individual $x_{i}$ in group i will produce in group j during its lifespan as infectious. We define group 0 as the entire population. For example, $R\left(x_{0},0\right)$ denotes the number of secondary infections that one infected individual $x_{0}$ (arbitrary in group 0, i.e., the entire population) will produce in the entire population during its lifespan as infectious. We use the tower property to compute the expectation of $R\left(x_{0},0\right)$:

(7) $$\begin{array}{l} R_{0}=E\left\{R\left(x_{0},0\right)\right\}\\ =E\left\{\overset{n}{\underset{j=1}{\sum }}R\left(x_{0},j\right)\right\}\\ =\overset{n}{\underset{j=1}{\sum }}E\left\{R\left(x_{0},j\right)\right\}\\ =\overset{n}{\underset{j=1}{\sum }}E_{R\left(x_{i},j\right),\;i=1,2,\cdots ,n}\left\{E\left\{R\left(x_{0},j\right)|R\left(x_{i},j\right),\;i=1,2,\cdots ,n\right\}\right\}\\ =\overset{n}{\underset{j=1}{\sum }}E_{R\left(x_{i},j\right),\;i=1,2,\cdots ,n}\left\{\overset{n}{\underset{i=1}{\sum }}P\left(x_{0}\in N_{i}\right)R\left(x_{i},j\right)\right\}\\ =\overset{n}{\underset{j=1}{\sum }}\overset{n}{\underset{i=1}{\sum }}P\left(x_{0}\in N_{i}\right)E_{R\left(x_{i},j\right)}\left\{R\left(x_{i},j\right)\right\}\\ =\overset{n}{\underset{j=1}{\sum }}\overset{n}{\underset{i=1}{\sum }}P\left(x_{0}\in N_{i}\right)R_{ij}\\ =\overset{n}{\underset{i=1}{\sum }}\left[P\left(x_{0}\in N_{i}\right)\overset{n}{\underset{j=1}{\sum }}R_{ij}\right] \end{array}$$

Here $P\left(x_{0}\in N_{i}\right)$ denote the probability of an arbitrarily selected individual $x_{0}$ belongs to group i, and it is determined by the proportion of the size of group i.

Random variable $R\left(x_{0},0\right)$ contains the stochasticity of $x_{0}$ may come from different groups and the stochasticity of $x_{0}$ may develop into different compartments. However, the random variable $R\left(x_{i},j\right)$ contains only the stochasticity that $x_{0}$ may develop into different compartments.

We can see that the expectation results in a weighted averaging of interactive $R_{ij}$.

#### 3.3.2 NGM for *R*_0_ (SDS)

In the NGM, $R_{0}$ is defined as the leading eigenvalue of the next-generation matrix M:

$$R_{0}=\lambda_{max}\left(M\right)$$

In most cases, the leading eigenvalue cannot be formulated as eigenvalues of submatrices $M_{ij}$, however, there are circumstances that $\lambda_{max}\left(M\right)$ still have a simple and comprehensive formulation. Assuming $f_{1}=f_{2}=\cdots =f_{n}=f$, then $V_{ii}$ is identical to $\;V_{11}$. Therefore:

$$M_{ij}=b_{ij}C,\;M=B⊗C$$

where ⊗ is Kronecker product, $b_{ij}=S_{i}\beta_{ji}$.

$$C=\left[\begin{array}{lll} \frac{1}{d_{r}+p\omega^{\text{′}}+\left(1-p\right)\omega }\left(\frac{\kappa p\omega^{\text{′}}}{\left(\gamma^{\text{′}}+d_{r}\right)}+\frac{\left(1-p\right)\omega }{\left(\gamma +d_{r}+f_{i}\right)}\right) & \frac{\kappa }{d_{r}+\gamma^{\text{′}}} & \frac{1}{d_{r}+f+\gamma }\\ 0 & 0 & 0\\ 0 & 0 & 0 \end{array}\right],$$

$$B=\left[\begin{array}{llll} b_{11} & b_{12} & \cdots & b_{1n}\\ b_{21} & b_{22} & \cdots & b_{2n}\\ \vdots & \vdots & \ddots & \vdots \\ b_{n1} & b_{n2} & \cdots & b_{nn} \end{array}\right]$$

Using properties of Kronecker product (Petersen & Pedersen, 2008), one obtains:

(8) $$\begin{array}{l} R_{0}=\lambda_{max}\left(M\right)\\ =\lambda_{max}\left(B\right)\lambda_{max}\left(C\right)\\ =\frac{\lambda_{max}\left(B\right)}{d_{r}+p\omega^{\text{′}}+\left(1-p\right)\omega }\left[\frac{\kappa p\omega^{\text{′}}}{\left(\gamma^{\text{′}}+d_{r}\right)}+\frac{\left(1-p\right)\omega }{\left(\gamma +d_{r}+f\right)}\right] \end{array}$$

We can see that, with the simplification that the natural death rates of each group are equal, the $R_{0}$ derived by NGM can be formulated as the eigenvalue of matrix of the transmission coefficient $\beta_{ij}$ multiplies a group-irrelevant coefficient, that is, the eigenvalue of the $R_{ij}$ matrix.

One may notice that NGM and DBM are both perform somehow ‘averaging’ to the interactive $R_{ij}$. The weighted averaging is adopted for DBM, while NGM averages $R_{ij}$ by taking leading eigenvalue.

### 3.4 R_0_ for SM

#### 3.4.1 Computing by DBM (SM)

The secondary infections produced by one infected individual during its infectious period can be divided into two parts: the secondary infection generated by environmental media that this infected individual produced, and the secondary infections generated by direct contact (no environmental media involved). We compute those two parts respectively and then take summation. The steps are as follows:

Step 1 Since P(x∈E)=1, then for all infected compartments, the probability that one infected individual x will attain is given by:

$$\begin{array}{cc} P\left(x\in A\right)=\;P\left(x\in A|x\in E\right)P\left(x\in E\right)= & \frac{p\omega^{\text{′}}}{p\omega^{\text{′}}+\left(1-p\right)\omega +d_{r}}\\ P\left(x\in I\right)=P\left(x\in I|x\in E\right)P\left(x\in E\right)= & \frac{\left(1-p\right)\omega_{1}}{p\omega^{\text{′}}+\left(1-p\right)\omega +d_{r}} \end{array}$$

Step 2 Letting (I,A)=(1,0) and (0,1) in the transmission rate βS(I+κA) respectively, one obtains the number of secondary infections that individual x will produce per unit time by direct contact:

$$Q_{1}\left(x\right)=\left\{ \begin{array}{cc} \kappa \beta S, & \;if\;x\in A\\ \beta S, & \;if\;x\in I \end{array}\right.$$

Step 3 The infectious period of x is given by:

$$T_{1}\left(x\right)=\left\{ \begin{array}{cc} 1/\left(\gamma^{\text{′}}+d_{r}\right), & \;if\;x\in A\\ 1/\left(\gamma +d_{r}\right), & \;if\;x\in I \end{array}\right.$$

Step 4 The secondary infections that individual x produced directly (without environmental media) during its lifespan as infectious are:

$$\begin{array}{ll} R_{1}=P\left(x\in A|x\in E\right)Q_{1}\left(x\in A\right)T_{1}\left(x\in A\right)+P\left(x\in I|x\in E\right)Q_{1}\left(x\in I\right)T_{1}\left(x\in I\right)= & \frac{\beta S}{p\omega^{\text{′}}+\left(1-p\right)\omega +d_{r}}\times \left[\frac{\kappa p\omega^{\text{′}}}{\gamma^{\text{′}}+d_{r}}+\frac{\left(1-p\right)\omega }{\gamma +d_{r}}\right] \end{array}$$

Step 5 The producing rate of infectious environmental media that individual x produces is:

$$l\left(x\right)=\left\{ \begin{array}{cc} \mu^{\text{′}}, & \;if\;x\in A\\ \mu , & \;if\;x\in I \end{array}\right.$$

And the amount of infectious environmental media that an individual x produced during its lifespan as infectious is:

$$L\left(x\right)=T\left(x\right)l\left(x\right)=\left\{ \begin{array}{cc} \mu^{\text{′}}/\left(\gamma^{\text{′}}+d_{r}\right), & \;if\;x\in A\\ \mu /\left(\gamma +d_{r}\right), & \;if\;x\in I \end{array}\right.$$

Step 6 Assuming that the current infectious environmental media is much greater than the amount that one individual can produce. This implies the infectivity of environmental media can be viewed as a constant for time interval T(x). Hence the secondary infections that L(x) produces can be computed by letting W=L(x) in $\beta_{W}SW$, and multiplies the inverse of removing rate 1/ε.

$$\begin{array}{ll} R_{2}=P\left(x\in A|x\in E\right)\beta_{W}S\frac{L\left(x\in A\right)}{\varepsilon }+P\left(x\in I|x\in E\right)\beta_{W}S\frac{L\left(x\in I\right)}{\varepsilon }= & \frac{p\mu^{\text{′}}\omega^{\text{′}}\beta_{W}S}{\varepsilon \left(\gamma^{\text{′}}+d_{r}\right)\left(p\omega^{\text{′}}+\left(1-p\right)\omega +d_{r}\right)}+\frac{\left(1-p\right)\mu \omega \beta_{W}S}{\varepsilon \left(\gamma +d_{r}\right)\left(p\omega^{\text{′}}+\left(1-p\right)\omega +d_{r}\right)} \end{array}$$

Step 7 Take the summation, the effective reproduction number is given by:

(9) $$\begin{array}{ll} R_{eff}=R_{1}+R_{2} & =\frac{\beta S}{p\omega^{\text{′}}+\left(1-p\right)\omega }\times \left[\frac{\kappa p\omega^{\text{′}}}{\gamma^{\text{′}}}+\frac{\left(1-p\right)\omega }{\gamma }\right]+\frac{p\mu^{\text{′}}\omega^{\text{′}}\beta_{W}S}{\varepsilon \left(\gamma^{\text{′}}+d_{r}\right)\left(p\omega^{\text{′}}+\left(1-p\right)\omega +d_{r}\right)}+\frac{\left(1-p\right)\mu \omega \beta_{W}S}{\varepsilon \left(\gamma +d_{r}\right)\left(p\omega^{\text{′}}+\left(1-p\right)\omega +d_{r}\right)} \end{array}$$

Step 8 $R_{0}$ is obtained by assuming that all individuals are susceptible, and substitutes S=N. Such a result is mutually equivalent to the result of NGM. (Derivation of NGM can be found in codes of supplementary materials)

### 3.5 Data validation for R_ij_ and R_0_

**Fig. 5:**
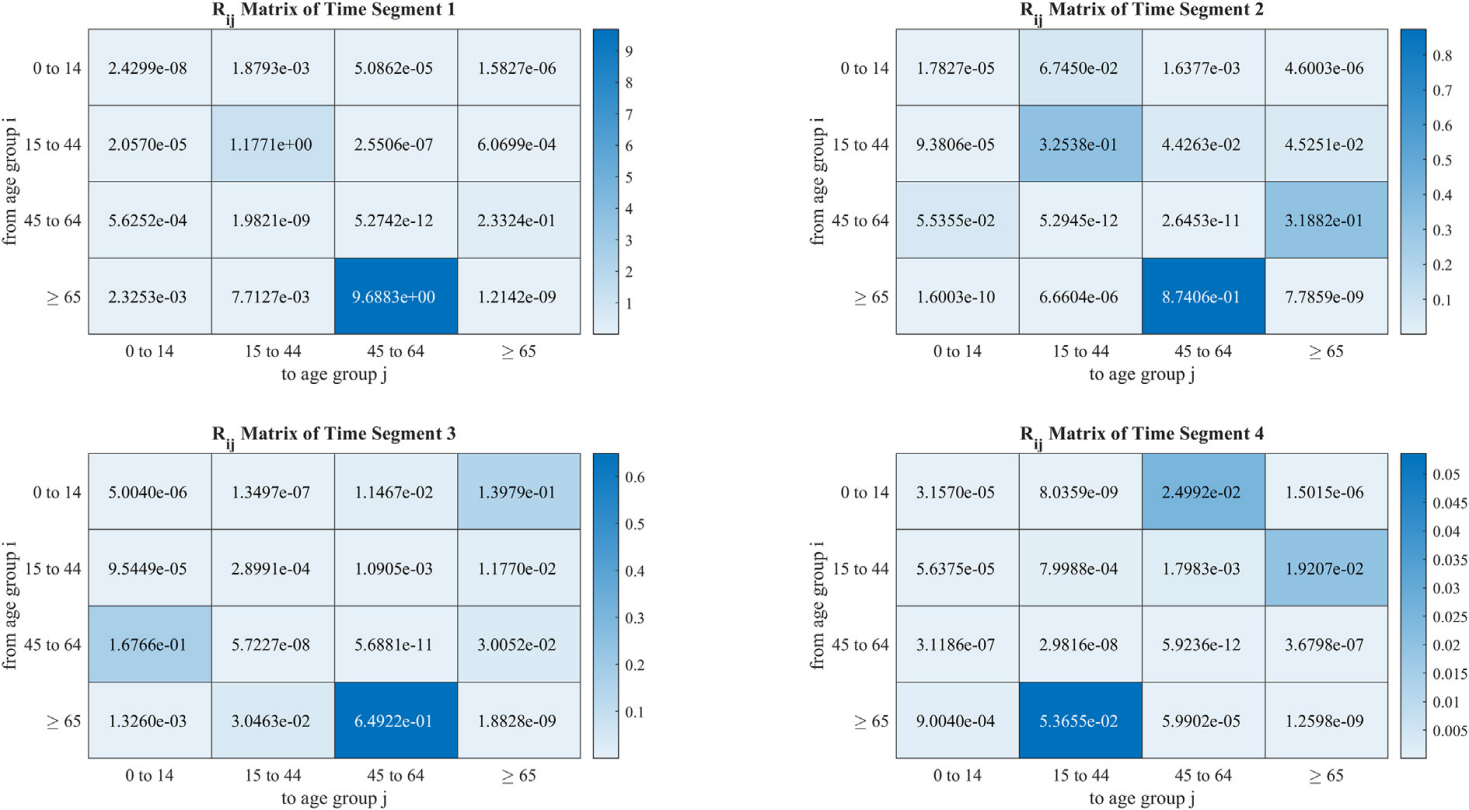
The *R*_*ij*_ matrices for four segments. The real-world COVID-19 data we used for validation was collected by Hunan Provincial CDC from January 5 to February 19, 2020. The data included patient gender, age, inter-provincial travel history, case type (symptomatic/asymptomatic), exposure date, date of onset, and date of diagnosis. Berkeley Madonna was employed to do the least root-mean-square deviation curve fitting. And for the sensitivity analysis, six parameters were employed to examine the model's sensitivity in this study, each of which was divided into 1000 values based on its range, mean and standard deviation data were calculated. The $R_{ij}$ Matrices for 4-time segments (defined as stages in the disease transmission period). Parameters for calculating are obtained from published research. Subfigure A represents time segment 1, from Jan 5, 2021 to Jan 25, 2021; subfigure B represents time segment 2, from Jan 25, 2021 to Jan 31, 2021; subfigure C represents time segment 3, from Jan 31, 2021 to Feb 5, 2021; subfigure D represents time segment 4, from Feb 5, 2021 to Feb 19, 2021.

According to the interactive $\beta_{ij}$ of 4 time-segments fitted on the basis of real-world COVID-19 data, the matrices of $R_{ij}$ of different time segments are evaluated by DBM in Fig. 5 (mutually equivalent to NGM). The $R_{0}$ for the entire population is also evaluated by two methods. DBM gives $R_{0}=1.7476$, while NGM gives $R_{0}=1.5032$. Such a difference is explained by the property of NGM: the further consideration of the transmissibility of secondary cases for continuous transmission makes result of NGM serves only as a threshold for the stability of disease-free equilibrium, and it is hard to explain in cases involving subgroups.

## 4 Discussion

DBM is formatted and generalized in the study, and further compared with NGM. It is found that: (i) For single-host dynamic models with a single group, results of DBM are identical to NGM. (ii) For single-host dynamic models with multi groups, the results of two methods in computing interactive *R*_*ij*_ are identical as well. However, in computing *R*_0_ for an entire population, the results differ, while DBM gives the true epidemiological interpretation.

Some studies adopted calculated *R*_0_ to estimate the transmissibility of infectious diseases, such as shigellosis (Z. Zhao, Chen, et al., 2021), HFMD (Xu et al., 2021), and COVID-19 (Zhao et al., 2021a). However, the detailed steps of deriving the *R*_0_ expression are not illustrated. Therefore, we described the derivations of DBM in detail, and extended the procedure to more complicated models, making computation of *R*_0_ accessible to the practitioners and researchers of public health. Also in these studies, only the parameters of the natural history of disease were included in dynamic models, whereas parameters of demographics were not considered. In this case, the calculated *R*_0_ is more suitable for an outbreak of infectious disease. When it comes to estimating the transmissibility of infectious disease through surveillance data of continuous years, however, the lack of demographic parameters could cause overestimation of true *R*_0_. This overestimation can act as a barrier against our understanding towards the transmission features of disease and cause excessive investments on the interventions. Hence, we developed DBM to compute *R*_0_ of single-host dynamic models, which parameters of demographic are sufficiently considered. The true *R*_0_ is therefore achievable both in outbreaks and regular preventions.

### 4.1 Comparison between DBM and NGM

The difference occurs while calculating *R*_0_ for the entire population with single-host dynamic models containing multi groups, by adopting DBM and NGM, respectively. Since the definition-based method uses the number of individuals in each subgroup as a proportion of the number of individuals in the entire host population to weight average $R_{ij}$, we can arbitrarily select group *i* to obtain the $R_{i}$ (expectation of secondary infectious that one infectious individual in subgroup *i* produces in the whole host population). In contrast, in the NGM, the *R*_0_ for the entire population is defined as the largest eigenvalue of the next-generation matrix and using the largest eigenvalue for ‘average’ has little epidemiological interpretation. Therefore, DBM is more suitable than NGM in the single-host dynamic models with multi groups.

### 4.2 Application in epidemics

In an epidemic outbreak, associated implications are urgently needed. If the data-driven approach of calculating *R*_0_ is adopted, it can take a long time to collect enough data, and sometimes *R*_0_ cannot be obtained until the end of the outbreak, for example, calculating *R*_0_ by the final size equation requires the proportion of susceptible individuals in the whole population at the end of the outbreak(Brauer, 2008). Whilst calculating *R*_0_ based on the dynamic compartment model, where some of the parameters can be obtained from a review of literature and others can be fitted to data from the early stages of an outbreak. This method allows for a rapid assessment of outbreak trends and informs the interventions required for control. Thus, the DBM of deriving *R*_0_ applies to many infectious disease outbreaks in which single-host dynamic models can be established correspondingly, such as influenza (Chen et al., 2017), COVID-19, Ebola (Chen et al., 2014, Chen et al., 2014), shigellosis, HFMD, etc. Besides, the derivation steps by DBM let practitioners gain a better understanding of the infectious disease dynamics. As we take apart the definition of *R*_0_, parameters that are essential in dynamic models may be plausible in interpreting public health interests. For example, the transmission rate and recovery rate determine overall speed and attack rate of epidemic, latent period determines duration of an epidemic. Interventions targeted at contact rate, infectious period and quarantine affect the transmission rate, recovery rate, and latent period, respectively (Ridenhour et al., 2018). The epidemiological application of *R*_0_ will only become valuable with accurate and cautious interpretations.

### 4.3 Other methods for reproduction number

There are three kinds of reproduction numbers: basic reproduction number *R*_0_, effective reproduction number *R_eff_*_,_ and time-varying or real-time reproduction number *R(t)*. Both *R*_0_ and *R_eff_* are functions of status (population status of entirely susceptible, non-intervention and social status of the certain economy and contact pattern for *R*_0_; specific status for *R_eff_*), while *R(t)* is a function of time. Once the status is expressed as functions of time (e.g., numerical solution of an ordinary differential equation), then we can immediately get *R(t)* from *R_eff_* by substitution. If the status at time 0 is simplified as entirely susceptible, non-intervention, then *R*_0_ can be obtained by letting t = 0 in *R(t),* i.e., *R(0).* Popular methods for time-varying *R(t)* use the distribution of generation time as a simplification of the natural history of disease and get free from the ordinary differential equations (Batista, 2021; Cori et al., 2013; Obadia et al., 2012; Thompson et al., 2019; Wallinga & Lipsitch, 2007). These methods are direct and fast implemented, while the resulting *R(0)* is only a reproduction number of initial time instance of an outbreak, and is not suitable to be interpreted as ‘basic’ reproduction number, especially with significant vaccination and NPIs (non-pharmacological interventions) at the initial time instance (the situation we faced in local outbreaks of Covid-19). A more reasonable way for computing *R*_0_ is to develop models with vaccination and intervention, and derive *R*_0_ from the calibrated and degenerated model (degenerate by letting the intensity of vaccination and intervention be zero).

### 4.4 Limitations

There are still some limitations of calculating *R*_0_ by DBM, which can only be applied to dynamic models with a single host population. For multi-host compartment models or co-dynamics models (Tchoumi et al., 2021), it is not able to establish a reasonable definition of *R*_0_, so the universal NGM is frequently used to get the threshold quantity of stability of disease-free equilibrium. In addition, in SIRC (C for chronic infection) models (Cui et al., 2020; Wang et al., 2020) for hepatitis C established by previous studies, the researchers used The Castillo-Chavez, Feng, and Huang approach of the next-generation matrix method to consider the changing of natural birth and death rates in susceptible populations over time, the result corresponds to making the susceptible population of *R*_*eff*_ obtained by the definition-based method equal to the extreme of the total population, which is more applicable for chronic infectious diseases. Yet if we use Van den Driessche and Watmough approach of the next-generation method (NGM) to calculate *R*_0_ in this case, the result is still equivalent to the definition-based method, if it is assumed that the natural birth and death rate are balanced, the results of the three methods are consistent. At the same time, because *R*_0_ itself has its limitations, model builders who want to compare the transmissibility between diseases based on *R*_0_ should also consider: Even for the same disease, there can be differences in population structure and social behavior in different periods and regions, adjustments should be made to the corresponding parameter assumptions (Delamater et al., 2019). Variables that affect contact patterns, such as birth rate, death rate, population density, cultural practices, etc., are usually similar within a region but vary in different areas (Guerra et al., 2017). Therefore, further studies might adopt more reasonable assumptions about the parameters in different regions at each stage if contact rate surveys are conducted in different regions at regular intervals.

## Funding

This study was partly supported by the 10.13039/100000865Bill & Melinda Gates Foundation (INV-005834), The 10.13039/501100012166National Key Research and Development Program of China (2021YFC2301604), the research project on education and teaching reform of undergraduate universities of Fujian Province, China (FBJG20210260).

## Author contributions

GXH, GYC, ZZY, and CTM conceived and designed the study; GXH, GYC, ZZY, YST, SYH, ZBH, and CTM conducted the analysis and the framework of the study; GXH and GYC wrote the manuscript; All authors contributed to revising subsequent versions of the manuscript. All authors read and approved the final manuscript.

## Declaration of interest

The authors declare that they have no competing interests.

## Declaration of interests

The authors declare that they have no known competing financial interests or personal relationships that could have appeared to influence the work reported in this paper.
