# Supplementary material for: Computing R0 of dynamic models by a definition-based method
Source: Infect Dis Model. 2022 May 24;7(2):196–210. doi: 10.1016/j.idm.2022.05.004 (PMC9160772; doi:10.1016/j.idm.2022.05.004)
Supplement: Multimedia component 1 [file mmc1.pdf]

Supplementary Material

March 13, 2022

1 Dynamical Models and Derivation of R_0

1.1 Dimensions of Variables and Parameters

Once the framework or the ODEs are modeled, there are some practically useful tips for determining physical dimension in models.

First, each compartments (S, I , R and so on) denote population size, and intuitively has dimension [individual]. Then, since each quantity denotes by arrows in framework of models will leads to a compartment i.e. of dimension [individual], by accumulation of time, the quantities of arrows are all of dimension [individual $\cdot$ time^{-1}].

One may easily determines dimensions of all variable and parameters through these two steps.

In models involve two or more host population (or with a compartment of reservoir), due to the different dimensions of host population (say, population of human may have a dimension of [person], while the population of ticks or mosquito may have a dimension the same as the Breteau Index or some density), the conditional of the initial value problem of ODEs is more likely to be illness and unstable.

Normalization of variables is a useful method to reduce the condition number of the initial value problem (i.e. improve the stability of the numerical methods for solving ODEs).

The method of normalizing will be introduced with the SEIARW model in section 1.6.

1.2 SIR Model

1.2.1 Flowchart (SIR)

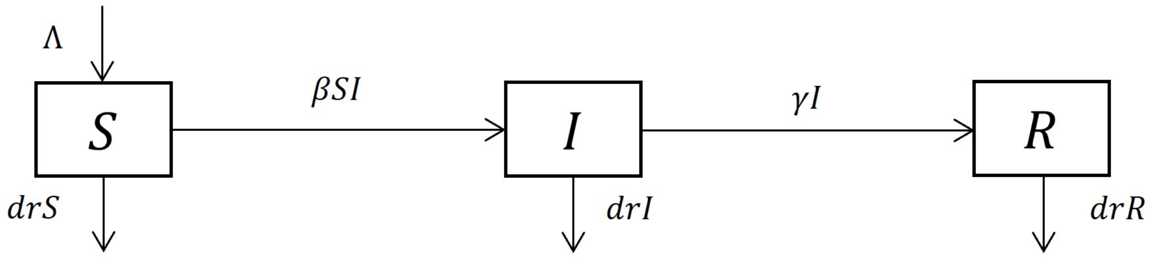

Fig. S 1: Flowchart of SIR Model. Variables S, I, R represent the susceptible, exposed, symptomatic infectious and recovered population; $N = S + I + R$ is size of entire population; Parameters Λ is the birth rate; dr denotes the mortality rate; β is the transmission rate coefficient; γ is the inverse of average infectious period for symptomatic cases.

1.2.2 Equations (SIR)

The corresponding ordinary differential equations are:

$$\frac{dS}{dt} = \Lambda - \beta SI - d_r S$$
$$\frac{dI}{dt} = \beta SI - \gamma I - d_r I$$
$$\frac{dR}{dt} = \gamma I - d_r R$$

The equation of total population $N = S + I + R$ is:

$$\frac{dN}{dt} = \Lambda - d_r N$$

The solution of this equation implies the population will converge to a constant Λ/d_r as time trend to infinity. If the method considers R_0 at disease free equilibrium, then $N = \Lambda/d_r$ will be substituted.

1.2.3 Variables and Parameters (SIR)

- S denotes the number of susceptible population
- I denotes the number of symptomatic infectious population
- R denotes the number of fully immunized (i.e. impossible to be infected) population
- $N = S + I + R$ is the total population size

- Λ denotes the birth rate
- d_r denotes the mortality rate coefficient
- β is newly infection rate coefficient
- γ is the inverse of average infectious period for symptomatic cases

1.2.4 DBM for SIR

(1) For one infected individual $x \in I$, let $I = 1$ in the newly infection term βSI in transition graph, one obtains the secondary infections produced by x per unit time:

$$Q(x \in I) = \beta S \tag{1}$$

(2) The removing rate γ is usually taken as the inverse of the average course of disease, that is, $1/\gamma$ is the average course of disease. By assuming the infectious period is equivalent to the average course of disease, by combining the terms γI and $d_r I$, the infectious period of x is given by:

$$T(x \in I) = \frac{1}{\gamma + d_r} \tag{2}$$

(3) Assuming the current cases are much smaller than the total population, and therefore, as a good 1-order approximation, we assume that the $Q(x \in I)$ remain constant during time interval $T(x \in I)$. Thus the secondary infections that x produced during its lifetime as infectious is:

$$R_{eff} = Q(x \in I)T(x \in I) \tag{3}$$

$$= \frac{\beta S}{\gamma + d_r} \tag{4}$$

(4) Assuming all individuals are susceptible and letting $S = N$, one obtains the R_0:

$$R_0 = \frac{\beta N}{\gamma + d_r} \tag{5}$$

Note that if the incident rate $\beta SI/N$ is used, then equation 1 changed to $Q(x \in I) = \beta S/N$, and thus DBM gives $R_0 = \frac{\beta}{\gamma + d_r}$.

1.2.5 NGM for SIR

(1) Split all infectious compartments (S,I,R) into to categories: the first (I) is infected, and the second (S,R) are not infected.

(2) Dividing the derivatives in differential equations of the first category (I) into two parts: the first $\mathscr{F}$ denote the

rate of newly infection, and the second $\mathscr{V}$ denote the transmission between infected compartments:

$$\frac{d}{dt}I = \beta SI - \gamma I - d_r I = \left[\beta SI\right] - \left[\gamma I + d_r I\right]$$
$$:= \mathscr{F} - \mathscr{V}$$

(3) Taking derivatives of $\mathscr{F}$ and $\mathscr{V}$ respect to I, one obtains the Jacobi matrix:

$$F = \beta S$$

$$V = \gamma + d_r$$

(4) R_{eff} is defined as the leading eigenvalue of the next generation matrix FV^{-1}, that is, $\beta S/(\gamma + d_r)$.

(5) Assuming that all individuals are susceptible, and substituting $S = N$, one obtains:

$$R_0 = \beta N/(\gamma + d_r)$$

Note that if the incident rate $\beta SI/N$ is used, $F = \beta S/N$, and thus NGM gives $R_0 = \frac{\beta}{\gamma + d_r}$, which is still identical to R_0 given by DBM.

1.3 SEIR

1.3.1 Flowchart (SIR)

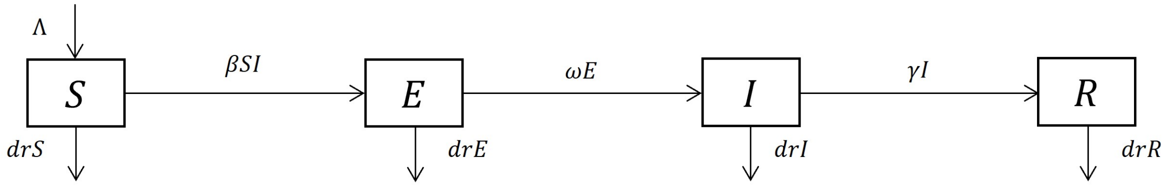

Fig. S 2: SEIR Model. Variables S, E, I, R represent the susceptible, exposed, symptomatic infectious and recovered population; $N = S + E + I + R$ is size of entire population; Parameters Λ is the birth rate; dr denotes the mortality rate; β is the transmission rate coefficient; ω is inverse of the average latent period of the symptomatic population, it is used to quantify the remove rate of compartment E (the inverse of incubation period is usually adopted for practice); γ is the inverse of average infectious period for symptomatic cases.

1.3.2 Equations (SIR)

The corresponding ordinary differential equations are:

$$\frac{dS}{dt} = \Lambda - \beta SI - d_r S$$
$$\frac{dE}{dt} = \beta SI - \omega E - d_r E$$
$$\frac{dI}{dt} = \omega E - \gamma I - d_r I$$
$$\frac{dR}{dt} = \gamma I - d_r R$$

The equation of total population $N = S + E + I + R$ is:

$$\frac{dN}{dt} = \Lambda - d_r N$$

The solution of this equation implies the population will converge to a constant Λ/d_r as time trend to infinity. If the method considers R_0 at disease free equilibrium, then $N = \Lambda/d_r$ will be substituted.

1.3.3 Variables and Parameters (SIR)

- S denotes the number of susceptible population
- E denotes the exposed population (infected but not yet infectious)
- I denotes the number of symptomatic infectious population
- R denotes the number of fully immunized (i.e. impossible to be infected) population
- $N = S + E + I + R$ is the total population size
- Λ denotes the birth rate
- d_r denotes the mortality rate coefficient
- β is newly infection rate coefficient
- ω is inverse of the average latent period of the symptomatic population, it is used to quantify the remove rate of compartment E. The inverse of incubation period is usually adopted for practice
- γ is the inverse of average infectious period for symptomatic cases

1.3.4 DBM for SEIR

(1) For one infected individual x, no matter whether $x \in I$ or $x \in E$ it is initially developed from state E. Thus we firstly consider compartment E. The probability that x attains compartment E and I are given:

$$\mathbb{P}(x \in E) = 1 \tag{6}$$

$$\mathbb{P}(x \in I) = \mathbb{P}(x \in I | x \in E)\mathbb{P}(x \in E) = \frac{\omega}{\omega + d_r} \tag{7}$$

(2) Letting $I = 1$, one obtains the secondary infection cased by $x \in I$ in unit time is:

$$Q(x \in I) = \beta S \tag{8}$$

(3) The infectious period is given by the average course of disease, together with natural death rate, the infectious period is given by:

$$T(x \in I) = \frac{1}{\gamma + d_r} \tag{9}$$

(4) Assuming the number of current cases is much smaller than the size of population, thus as a good approximation, we assume that $Q(x \in I)$ remain constant during time interval $T(x \in I)$. Therefore the secondary infections x will produce during its lifetime as infectious is given:

$$R_{eff} = Q(x \in I)T(x \in I) \tag{10}$$

$$= \frac{\omega \beta S}{(\omega + d_r)(\gamma + d_r)} \tag{11}$$

(5) Letting $S = N$, that is, all individuals are susceptible, an one obtains R_0:

$$R_0 = \frac{\omega \beta N}{(\omega + d_r)(\gamma + d_r)} \tag{12}$$

1.3.5 NGM for SEIR

(1) Divide (S,E,I,R) into two categories: the first (E,I) are infected, and the second (S,R) are noninfected.

(2) Divide the derivative of (E,I) into two parts: the first $\mathscr{F}$ represents rates of newly infections, and the second $\mathscr{V}$ represents rates of transition between (E,I):

$$\frac{d}{dt}\begin{bmatrix} E \\ I \end{bmatrix} = \begin{bmatrix} \beta SI - \omega E - d_r E \\ \omega E - \gamma I - d_r I \end{bmatrix} = \begin{bmatrix} \beta SI \\ 0 \end{bmatrix} - \begin{bmatrix} \omega E + d_r E \\ -\omega E + \gamma I + d_r I \end{bmatrix} \tag{13}$$

$$:= \mathscr{F} - \mathscr{V} \tag{14}$$

(3) Taking derivatives for $\mathscr{F}$ and $\mathscr{V}$ respect to (E,I), the Jacobi matrices are obtained:

$$F = \begin{bmatrix} 0 & \beta S \\ 0 & 0 \end{bmatrix}, \quad V = \begin{bmatrix} \omega + d_r & 0 \\ -\omega & \gamma + d_r \end{bmatrix} \tag{15}$$

the inverse of matrix V is further computed:

$$V^{-1} = \begin{pmatrix} \frac{1}{\mathrm{dr}+\omega} & 0 \\ \frac{\omega}{(\mathrm{dr}+\gamma)\,(\mathrm{dr}+\omega)} & \frac{1}{\mathrm{dr}+\gamma} \end{pmatrix}$$

(4) R_{eff} is defined as the leading eigenvalue of FV^{-1}:

$$FV^{-1} = \begin{pmatrix} \frac{S\,\beta\,\omega}{(\mathrm{dr}+\gamma)\,(\mathrm{dr}+\omega)} & \frac{S\,\beta}{\mathrm{dr}+\gamma} \\ 0 & 0 \end{pmatrix}$$

$$\lambda_{max}(FV^{-1}) = \frac{\omega\beta S}{(\omega + d_r)(\gamma + d_r)}$$

(5) Since R_0 is defined for the entirely susceptible population, hence $S = N$ is substituted, and one obtains:

$$R_0 = \frac{\omega\beta N}{(\omega + d_r)(\gamma + d_r)}$$

1.4 SEIAR

1.4.1 Flowchart (SEIAR)

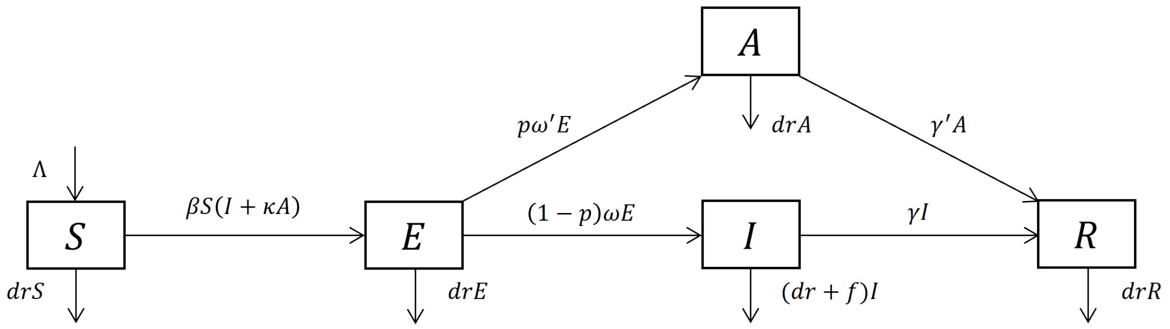

Fig. S 3: SEIAR Model. Variables S, E, I, A, R represent the susceptible, exposed, symptomatic infectious, asymptomatic infectious and recovered population; $N = S + E + I + A + R$ is size of entire population; Parameters Λ is the birth rate; dr denotes the mortality rate; f is the case fatality rate; β is the transmission rate coefficient; κ is the relatively of transmission ability of asymptomatic cases compared with the symptomatic cases; p is the probability that one infected individual will developed into a asymptomatic case; ω is inverse of the average latent period of the symptomatic population, it is used to quantify the remove rate of compartment E (the inverse of incubation period is usually adopted for practice); $\omega^{'}$ is the inverse of the average latent period of the asymptomatic population; γ is the inverse of average infectious period for symptomatic cases; $\gamma^{'}$ is the inverse of average infectious period for asymptomatic.

1.4.2 Equations (SEIAR)

The corresponding ordinary differential equations are:

$$\frac{dS}{dt} = \Lambda - \beta S(I + \kappa A) - d_r S$$
$$\frac{dE}{dt} = \beta S(I + \kappa A) - (1-p)\omega E - p\omega' E - d_r E$$
$$\frac{dI}{dt} = (1-p)\omega E - \gamma I - (d_r + f)I$$
$$\frac{dA}{dt} = p\omega' E - \gamma' A - d_r A$$
$$\frac{dR}{dt} = \gamma I + \gamma' A - d_r R$$

The equation of total population $N = S + E + I + A + R$ is:

$$\frac{dN}{dt} = \Lambda - d_r N$$

The solution of this equation implies the population will converge to a constant Λ/d_r as time trend to infinity. If the method considers R_0 at disease free equilibrium, then $N = \Lambda/d_r$ will be substituted.

1.4.3 Variables and Parameters (SEIAR)

- S denotes the number of susceptible population
- E denotes the exposed population (infected but not yet infectious)
- I denotes the number of symptomatic infectious population
- A denotes the number of asymptomatic infectious population
- R denotes the number of fully immunized (i.e. impossible to be infected) population
- $N = S + E + I + A + R$ is the total population size
- Λ denotes the birth rate
- d_r denotes the mortality rate coefficient
- β is newly infection rate coefficient
- κ is the relatively of transmission ability of asymptomatic cases compared with the symptomatic cases
- f is the case fatality rate
- p is the probability that one infected individual will developed into a asymptomatic case

- ω is inverse of the average latent period of the symptomatic population, it is used to quantify the remove rate of compartment E. The inverse of incubation period is usually adopted for practice
- $\omega_i^{'}$ is the inverse of the average latent period of the asymptomatic population
- γ is the inverse of average infectious period for symptomatic cases
- $\gamma_i^{'}$ is the inverse of average infectious period for asymptomatic cases

1.4.4 DBM for SEIAR

(1) For any infected individual x, it is initially developed into E. Thus our discussion start from E. The probabilities that x attains each infectious compartments are:

$$\mathbb{P}(x \in A) = \mathbb{P}(x \in A|x \in E)\mathbb{P}(x \in E) = \frac{p\omega^{'}}{p\omega^{'} + (1-p)\omega + d_r}$$
$$\mathbb{P}(x \in I) = \mathbb{P}(x \in I|x \in E)\mathbb{P}(x \in E) = \frac{(1-p)\omega}{p\omega^{'} + (1-p)\omega + d_r}$$

where $\mathbb{P}(x \in I|x \in E)$ denote the conditional probability of $x \in I$ when $x \in E$ known.
(2) Letting one of components of $(d_r E_i, I_i, A_i)$ equals to 1 and others equal to 0, and substitute in the newly infection term $\beta S(I + \kappa A)$, one obtains the secondary infection that x will produces in unit time in different states:

$$Q(x) = \begin{cases} 0, & \text{if } x \in d_r E \quad (dead) \\ \beta\kappa S, & \text{if } x \in A \\ \beta S, & \text{if } x \in I \end{cases}$$

(3) The infectious period is given by:

$$T(x) = \begin{cases} 0, & \text{if } x \in dE \quad (dead) \\ 1/(\gamma^{'} + d_r), & \text{if } x \in A \\ 1/(f + \gamma + d_r), & \text{if } x \in I \end{cases}$$

(4) At the beginning of disease, $S \approx N$. As a good approximation, it is natural to assume tha $Q(x)$ remain constant during time interval $T(x)$. Therefore, the secondary infections that x will produce during its lifespan as infectious is given by:

$$\begin{aligned} R_{eff} = &\mathbb{P}(x \in d_r E) \times 0 \times 0 \\ &+ \mathbb{P}(x \in A) \times \beta\kappa S \times (1/(\gamma^{'} + d_r)) \\ &+ \mathbb{P}(x \in I) \times \beta S \times (1/(f + \gamma + d_r)) \end{aligned}$$

(5) Assuming all individuals are susceptible, and substituting $S = N$, one obtains:

$$R_0 = \frac{\beta N}{d_r + p\omega' + (1-p)\omega}\left[\frac{\kappa p \omega^{'}}{(\gamma' + d_r)} + \frac{(1-p)\omega}{(\gamma + d_r + f)}\right]$$

1.4.5 NGM for SEIAR

(1) Divide (S,E,I,A,R) into two categories: the first (E,I,A) are infected, and the second (S,R) are noninfected.

(2) Divide the derivative of (E,I,A) into two parts: the first $\mathscr{F}$ represents rates of newly infections, and the second $\mathscr{V}$ represents rates of transition between (E,I,A):

$$\frac{d}{dt}\begin{bmatrix} E \\ I \\ A \end{bmatrix} = \begin{bmatrix} \beta S(I+\kappa A) - p\omega_2 E - (1-p)\omega_1 E - d_r E \\ (1-p)\omega_1 E - (f+\gamma)I - d_r I \\ p\omega_2 E - \gamma^{'} A - d_r A \end{bmatrix} = \begin{bmatrix} \beta S(I+\kappa A) \\ 0 \\ 0 \end{bmatrix} - \begin{bmatrix} p\omega_2 E + (1-p)\omega_1 E + d_r E \\ -(1-p)\omega_1 E + (f+\gamma)I + d_r I \\ -p\omega_2 E + \gamma^{'} A + d_r A \end{bmatrix}$$

$$:= \mathscr{F} - \mathscr{V}$$

(3) Take derivatives of the vector-valued function $\mathscr{F}$ and $\mathscr{V}$ respect to (E, I, A), the Jacobi matrices are obtained:

$$F = \begin{pmatrix} 0 & S\,\beta\,\kappa & S\,\beta \\ 0 & 0 & 0 \\ 0 & 0 & 0 \end{pmatrix}$$

$$V = \begin{pmatrix} d_r + \omega^{'}\,p - \omega\,(p-1) & 0 & 0 \\ -\omega^{'}\,p & d_r + \gamma^{'} & 0 \\ \omega\,(p-1) & 0 & d_r + f + \gamma \end{pmatrix}$$

the inverse of V is further computed:

$$V^{-1} = \begin{pmatrix} \frac{1}{d_r+\omega-\omega\,p+\omega^{'}\,p} & 0 & 0 \\ \frac{\omega^{'}\,p}{\left(d_r+\gamma^{'}\right)\left(d_r+\omega-\omega\,p+\omega^{'}\,p\right)} & \frac{1}{d_r+\gamma^{'}} & 0 \\ -\frac{\omega\,(p-1)}{(d_r+f+\gamma)\left(d_r+\omega-\omega\,p+\omega^{'}\,p\right)} & 0 & \frac{1}{d_r+f+\gamma} \end{pmatrix}$$

(4) Construct the next generation matrix $M = FV^{-1}$

$$M = \begin{pmatrix} \frac{S\,\beta\,\kappa\,\omega^{'}\,p}{\left(d_r+\gamma^{'}\right)\left(d_r+\omega-\omega\,p+\omega^{'}\,p\right)} - \frac{S\,\beta\,\omega\,(p-1)}{(d_r+f+\gamma)\left(d_r+\omega-\omega\,p+\omega^{'}\,p\right)} & \frac{S\,\beta\,\kappa}{d_r+\gamma^{'}} & \frac{S\,\beta}{d_r+f+\gamma} \\ 0 & 0 & 0 \\ 0 & 0 & 0 \end{pmatrix}$$

the leading eigenvalue:

$$\lambda_{max}(FV^{-1}) = \frac{S\,\beta\,\left(d_r\,\omega + \gamma^{'}\,\omega - d_r\,\omega\,p - \gamma^{'}\,\omega\,p + d_r\,\kappa\,\omega^{'}\,p + f\,\kappa\,\omega^{'}\,p + \gamma\,\kappa\,\omega^{'}\,p\right)}{(d_r + \gamma^{'})\,(d_r + f + \gamma)\,(d_r + \omega - \omega\,p + \omega^{'}\,p)}$$

(5) Substitute the disease-free equilibrium (S,E,A,I,R) = (N,0,0,0,0), one obtains:

$$R_0 = \frac{S\,\beta\,\left(d_r\,\omega + \gamma^{'}\,\omega - d_r\,\omega\,p - \gamma^{'}\,\omega\,p + d_r\,\kappa\,\omega^{'}\,p + f\,\kappa\,\omega^{'}\,p + \gamma\,\kappa\,\omega^{'}\,p\right)}{(d_r + \gamma')\,(d_r + f + \gamma)\,(d_r + \omega - \omega\,p + \omega'\,p)}$$

by few steps of simplification, one can see that this R_0 is identical to the result of DBM.

1.5 SIRC Model

1.5.1 Flowchart (SIRC)

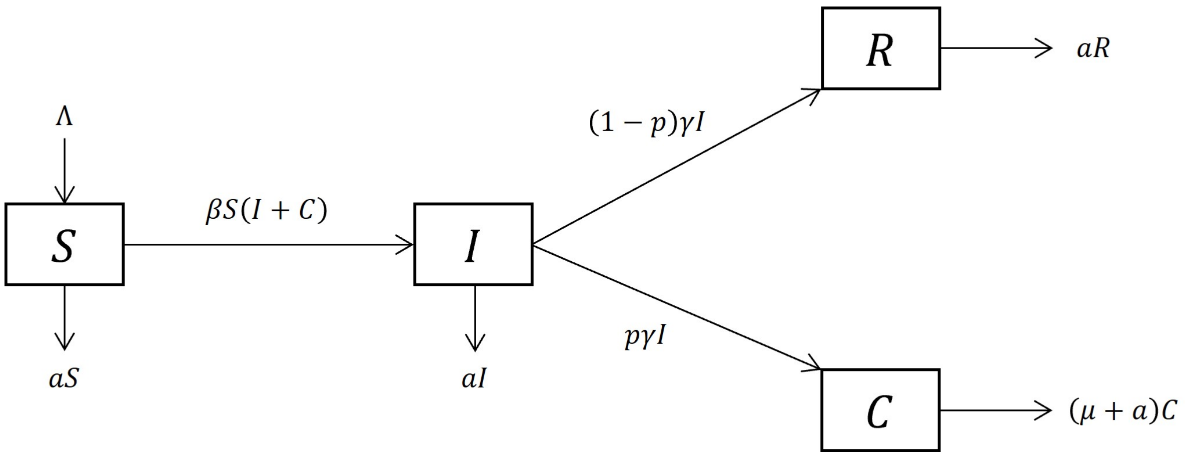

Fig. S 4: Flowchart of SIRC Model. Variables S, I, R, C represent population of susceptible, acute infected, recovered and chronic infected. Parameters Λ represent birth rate; β is the transmission rate coefficient; a is the natural death rate; μ is the fatality; p represent the proportion of progressing to chronic stage; γ is the inverse of the duration of mean acute infection

1.5.2 Equations (SIRC)

The corresponding ordinary differential equations are:

$$\frac{dS}{dt} = \Lambda - \beta S(I + C) - aS$$
$$\frac{dI}{dt} = \beta S(I + C) - \gamma I - aI$$
$$\frac{dR}{dt} = (1 - p)\gamma I - aR$$
$$\frac{dC}{dt} = p\gamma I - (\mu + a)C$$

The disease-free equilibrium can be solved from:

$$\frac{dN}{dt} = \Lambda - d_r N$$

where $N = S + I + R + C$

The solution of this equation implies the population will converge to a constant Λ/d_r as time trend to infinity, if the population is disease-free. If the method considers R_0 at disease free equilibrium, then $N = \Lambda/d_r$ will be substituted.

1.5.3 Variables and Parameters (SIRC)

- S denotes the number of susceptible population
- I denotes the number of symptomatic infectious population
- R denotes the number of fully immunized (i.e. impossible to be infected) population
- C denotes the number of chronic infected populations (infectious)
- $N = S + I + R + C$ is the total population size
- Λ denotes the birth rate
- a denotes the mortality rate coefficient
- μ is the chronic death rate coefficient
- β is newly infection rate coefficient
- γ is the inverse of average infectious period for symptomatic cases
- p is the proportion of progressing to chronic stage

1.5.4 Derivation of R_0 (SIRC)

The definition-based method (DBM) is implemented in the main text as an example for introducing general statement of DBM.

The next-generation method of this model is quiet similar to the previous derivation, and can be found in MATLAB codes of next section.

1.6 SEIARW Model

1.6.1 Flowchart (SEIARW)

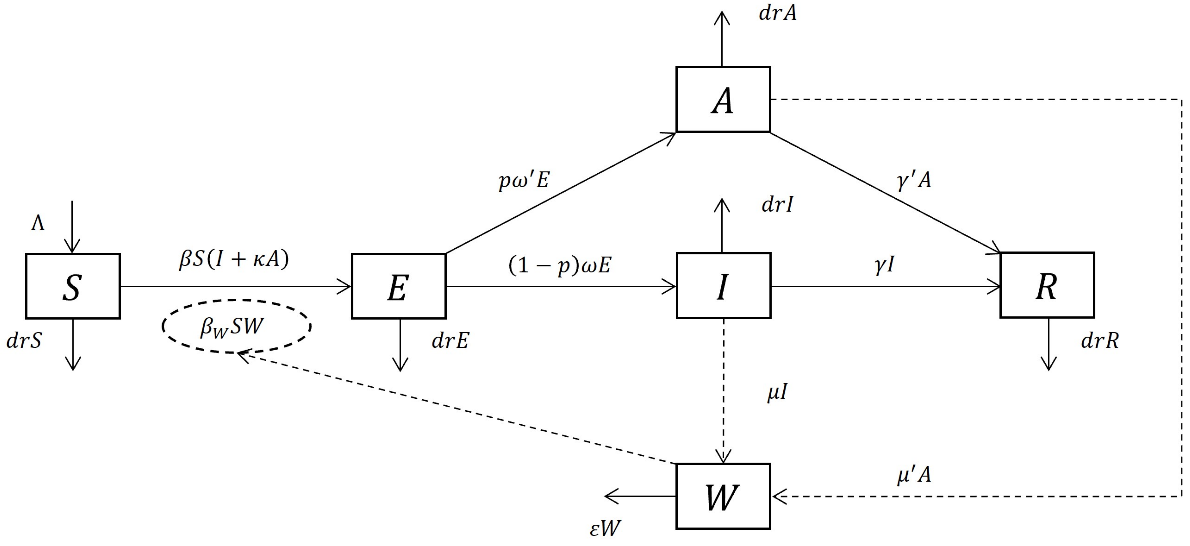

Fig. S 5: Flowchart of SEIARW Model. Variables S, E, I, A, R, W represent population of susceptible, exposed, symptomatic infectious, asymptomatic infectious, recovered and pathogen in reservoir. Parameters Λ is the birth rate; β is the human-to-human transmission rate coefficient; β_W is the reservoir-to-human transmission rate coefficient; κ is the relatively of transmission ability of asymptomatic cases compared with the symptomatic cases; d_r is the natural death rate; f is the fatality; p represent the proportion of progressing to asymptomatic infectious stage; ω is the inverse of the average latent period of the symptomatic cases; $\omega^{'}$ is the inverse of the average latent period of the asymptomatic cases; γ is the inverse of the infectious period of symptomatic cases; $\gamma^{'}$ is the inverse of average infectious period of asymptomatic cases.

1.6.2 Equations (SEIARW)

The corresponding ordinary differential equations are:

$$\frac{dS}{dt} = \Lambda - \beta S(I + \kappa A) - \beta_W SW - d_r S$$
$$\frac{dE}{dt} = \beta S(I + \kappa A) + \beta_W SW - (1-p)\omega E - p\omega^{'} E - d_r E$$
$$\frac{dI}{dt} = (1-p)\omega E - \gamma I - (d_r + f)I$$
$$\frac{dA}{dt} = p\omega^{'} E - \gamma^{'} A - d_r A$$
$$\frac{dR}{dt} = \gamma I + \gamma^{'} A - d_r R$$
$$\frac{dW}{dt} = \mu I + \mu^{'} A - \epsilon W$$

1.6.3 Variables and Parameters (SEIARW)

- S denotes the number of susceptible population
- E denotes the exposed population (infected but not yet infectious)
- I denotes the number of symptomatic infectious population
- A denotes the number of asymptomatic infectious population
- R denotes the number of fully immunized (i.e. impossible to be infected) population
- W denotes pathogen in reservoir (water, air and so on)
- $N = S + E + I + A + R$ is the total population size
- Λ denotes the birth rate
- d_r denotes the mortality rate coefficient
- β is newly infection rate coefficient through contact of infected individuals
- β_W is newly infection rate coefficient through reservoir
- κ is the relatively of transmission ability of asymptomatic cases compared with the symptomatic cases
- f is the case fatality rate
- p is the probability that one infected individual will developed into a asymptomatic case
- ω is inverse of the average latent period of the symptomatic population, it is used to quantify the remove rate of compartment E. The inverse of incubation period is usually adopted for practice
- $\omega_i^{'}$ is the inverse of the average latent period of the asymptomatic population
- γ is the inverse of average infectious period of symptomatic cases
- $\gamma_i^{'}$ is the inverse of average infectious period of asymptomatic cases
- μ is the rate coefficient that symptomatic cases produce pathogens to reservoir
- $\mu^{'}$ is the rate coefficient that asymptomatic cases produce pathogens to reservoir
- ϵ is the decay rate of pathogen in reservoir coefficient

1.6.4 Derivation of R_0 (SEIARW)

The definition-based method (DBM) is implemented in the main text to prove the effectiveness of DBM.

The steps of next-generation method of this model is listed as follows. Here MATLAB is used to assist the computation. Using the symbolic toolbox and use latex() function to transform the symbolic expressions into its latex codes. Then one can use pandoc or mathpix to transform the latex codes into Microsoft Word formulas. The Matlab codes are listed with a link for download in the last chapter of this supplementary material.

For convenience in Matlab coding, we shall used γ_1, γ_2 instead of γ, $\gamma^{'}$, and similar for other parameters.

NGM:

(1) Divide (S,E,I,A,R,W) into two categories: the first (E,I,A,W) are infected, and the second (S,R) are non-infected.

(2) Divide the derivative of (E,I,A,W) into two parts: the first $\mathscr{F}$ represents rates of newly infections, and the second $\mathscr{V}$ represents rates of transition between (E,I,A,W):

$$\frac{d}{dt}\begin{bmatrix} E \\ I \\ A \\ W \end{bmatrix} = \begin{bmatrix} S\,W\,\beta_w - E\,\mathrm{dr} - E\,\omega_2\,p + S\,\beta\,\left(A\,\kappa + I\right) + E\,\omega_1\,\left(p-1\right) \\ -I\,\mathrm{dr} - I\,\gamma_1 - E\,\omega_1\,\left(p-1\right) \\ -A\,\mathrm{dr} - A\,\gamma_2 + E\,\omega_2\,p \\ -W\,\epsilon + I\,\mu_1 + A\,\mu_2 \end{bmatrix} \tag{16}$$

$$= \begin{bmatrix} S\,W\,\beta_w + S\,\beta\,\left(A\,\kappa + I\right) \\ 0 \\ 0 \\ 0 \end{bmatrix} - \begin{bmatrix} E\,\mathrm{dr} + E\,\omega_2\,p - E\,\omega_1\,\left(p-1\right) \\ I\,\mathrm{dr} + I\,\gamma_1 + E\,\omega_1\,\left(p-1\right) \\ A\,\mathrm{dr} + A\,\gamma_2 - E\,\omega_2\,p \\ W\,\epsilon - I\,\mu_1 - A\,\mu_2 \end{bmatrix} \tag{17}$$

$$:= \mathscr{F} - \mathscr{V} \tag{18}$$

(3) Taking derivatives for $\mathscr{F}$ and $\mathscr{V}$ respect to (E,I), the Jacobi matrices are obtained:

$$F = \begin{bmatrix} 0 & S\,\beta & S\,\beta\,\kappa & S\,\beta_w \\ 0 & 0 & 0 & 0 \\ 0 & 0 & 0 & 0 \\ 0 & 0 & 0 & 0 \end{bmatrix}, \quad V = \begin{bmatrix} \mathrm{dr} + \omega_2\,p - \omega_1\,\left(p-1\right) & 0 & 0 & 0 \\ \omega_1\,\left(p-1\right) & \mathrm{dr} + \gamma_1 & 0 & 0 \\ -\omega_2\,p & 0 & \mathrm{dr} + \gamma_2 & 0 \\ 0 & -\mu_1 & -\mu_2 & \epsilon \end{bmatrix} \tag{19}$$

the inverse of matrix V is further computed:

$$V^{-1} = \begin{bmatrix} \frac{1}{\mathrm{dr}-\omega_1\, p+\omega_1+\omega_2\, p} & 0 & 0 & 0 \\ -\frac{\omega_1\, (p-1)}{(\mathrm{dr}+\gamma_1)\, (\mathrm{dr}-\omega_1\, p+\omega_1+\omega_2\, p)} & \frac{1}{\mathrm{dr}+\gamma_1} & 0 & 0 \\ \frac{\omega_2\, p}{(\mathrm{dr}+\gamma_2)\, (\mathrm{dr}-\omega_1\, p+\omega_1+\omega_2\, p)} & 0 & \frac{1}{\mathrm{dr}+\gamma_2} & 0 \\ \frac{-\mathrm{dr}\, \mu_1\, \omega_1\, p+\mathrm{dr}\, \mu_1\, \omega_1+\mathrm{dr}\, \mu_2\, \omega_2\, p+\gamma_1\, \mu_2\, \omega_2\, p-\gamma_2\, \mu_1\, \omega_1\, p+\gamma_2\, \mu_1\, \omega_1}{\epsilon\, (\mathrm{dr}+\gamma_1)\, (\mathrm{dr}+\gamma_2)\, (\mathrm{dr}-\omega_1\, p+\omega_1+\omega_2\, p)} & \frac{\mu_1}{\epsilon\, (\mathrm{dr}+\gamma_1)} & \frac{\mu_2}{\epsilon\, (\mathrm{dr}+\gamma_2)} & \frac{1}{\epsilon} \end{bmatrix}$$

(4) R_{eff} is defined as the leading eigenvalue of FV^{-1}:

$$FV^{-1} = \begin{bmatrix} M_{11} & \frac{S\,\beta}{\mathrm{dr}+\gamma_1} + \frac{S\,\beta_w\,\mu_1}{\epsilon\,(\mathrm{dr}+\gamma_1)} & \frac{S\,\beta\,\kappa}{\mathrm{dr}+\gamma_2} + \frac{S\,\beta_w\,\mu_2}{\epsilon\,(\mathrm{dr}+\gamma_2)} & \frac{S\,\beta_w}{\epsilon} \\ 0 & 0 & 0 & 0 \\ 0 & 0 & 0 & 0 \\ 0 & 0 & 0 & 0 \end{bmatrix}$$

where

$$M_{11} = \frac{S\,\beta_w\; (-\mathrm{dr}\,\mu_1\,\omega_1\,p + \mathrm{dr}\,\mu_1\,\omega_1 + \mathrm{dr}\,\mu_2\,\omega_2\,p + \gamma_1\,\mu_2\,\omega_2\,p - \gamma_2\,\mu_1\,\omega_1\,p + \gamma_2\,\mu_1\,\omega_1)}{\epsilon\; (\mathrm{dr}+\gamma_1)\; (\mathrm{dr}+\gamma_2)\; (\mathrm{dr}-\omega_1\,p+\omega_1+\omega_2\,p)} \tag{20}$$

$$-\frac{S\,\beta\,\omega_1\;(p-1)}{(\mathrm{dr}+\gamma_1)\;(\mathrm{dr}-\omega_1\,p+\omega_1+\omega_2\,p)} + \frac{S\,\beta\,k\,\omega_2\,p}{(\mathrm{dr}+\gamma_2)\;(\mathrm{dr}-\omega_1\,p+\omega_1+\omega_2\,p)} \tag{21}$$

The maximum real part of eigenvalues:

$$\lambda_{max}(FV^{-1}) = \frac{S\,\beta_w\; (-\mathrm{dr}\,\mu_1\,\omega_1\,p + \mathrm{dr}\,\mu_1\,\omega_1 + \mathrm{dr}\,\mu_2\,\omega_2\,p + \gamma_1\,\mu_2\,\omega_2\,p - \gamma_2\,\mu_1\,\omega_1\,p + \gamma_2\,\mu_1\,\omega_1)}{\epsilon\; (\mathrm{dr}+\gamma_1)\; (\mathrm{dr}+\gamma_2)\; (\mathrm{dr}-\omega_1\,p+\omega_1+\omega_2\,p)} \tag{22}$$

$$-\frac{S\,\beta\,\omega_1\;(p-1)}{(\mathrm{dr}+\gamma_1)\;(\mathrm{dr}-\omega_1\,p+\omega_1+\omega_2\,p)} + \frac{S\,\beta\,\kappa\,\omega_2\,p}{(\mathrm{dr}+\gamma_2)\;(\mathrm{dr}-\omega_1\,p+\omega_1+\omega_2\,p)} \tag{23}$$

(5) Since R_0 is defined for the entirely susceptible population, hence $S = N$ is substituted, and one obtains:

$$R_0 = \frac{N\,\beta_w\; (-\mathrm{dr}\,\mu_1\,\omega_1\,p + \mathrm{dr}\,\mu_1\,\omega_1 + \mathrm{dr}\,\mu_2\,\omega_2\,p + \gamma_1\,\mu_2\,\omega_2\,p - \gamma_2\,\mu_1\,\omega_1\,p + \gamma_2\,\mu_1\,\omega_1)}{\epsilon\; (\mathrm{dr}+\gamma_1)\; (\mathrm{dr}+\gamma_2)\; (\mathrm{dr}-\omega_1\,p+\omega_1+\omega_2\,p)} \tag{24}$$

$$-\frac{N\,\beta\,\omega_1\;(p-1)}{(\mathrm{dr}+\gamma_1)\;(\mathrm{dr}-\omega_1\,p+\omega_1+\omega_2\,p)} + \frac{N\,\beta\,\kappa\,\omega_2\,p}{(\mathrm{dr}+\gamma_2)\;(\mathrm{dr}-\omega_1\,p+\omega_1+\omega_2\,p)} \tag{25}$$

$$\tag{26}$$

$$= \frac{N\,\beta_w\; [p\mu_2\omega_2(dr+\gamma_1) + (1-p)\mu_1\omega_1(dr+\gamma_2)]}{\epsilon\; (\mathrm{dr}+\gamma_1)\; (\mathrm{dr}+\gamma_2)\; (\mathrm{dr}-\omega_1\,p+\omega_1+\omega_2\,p)} \tag{27}$$

$$-\frac{N\,\beta\,\omega_1\;(p-1)}{(\mathrm{dr}+\gamma_1)\;(\mathrm{dr}-\omega_1\,p+\omega_1+\omega_2\,p)} + \frac{N\,\beta\,\kappa\,\omega_2\,p}{(\mathrm{dr}+\gamma_2)\;(\mathrm{dr}-\omega_1\,p+\omega_1+\omega_2\,p)} \tag{28}$$

$$\tag{29}$$

$$= \frac{N\,\beta_w\,p\mu_2\omega_2}{\epsilon\,(\mathrm{dr}+\gamma_2)\;(\mathrm{dr}-\omega_1\,p+\omega_1+\omega_2\,p)} + \frac{N\,\beta_w(1-p)\mu_1\omega_1}{\epsilon\,(\mathrm{dr}+\gamma_1)\;(\mathrm{dr}-\omega_1\,p+\omega_1+\omega_2\,p)} \tag{30}$$

$$-\frac{N\,\beta\,\omega_1\;(p-1)}{(\mathrm{dr}+\gamma_1)\;(\mathrm{dr}-\omega_1\,p+\omega_1+\omega_2\,p)} + \frac{N\,\beta\,\kappa\,\omega_2\,p}{(\mathrm{dr}+\gamma_2)\;(\mathrm{dr}-\omega_1\,p+\omega_1+\omega_2\,p)} \tag{31}$$

One can see that the right hand side of the last equation is mutually equivalent to result of DBM in the main text.

1.7 Multi-Group SEIAR Model

1.7.1 Flowchart (multi-group SEIAR)

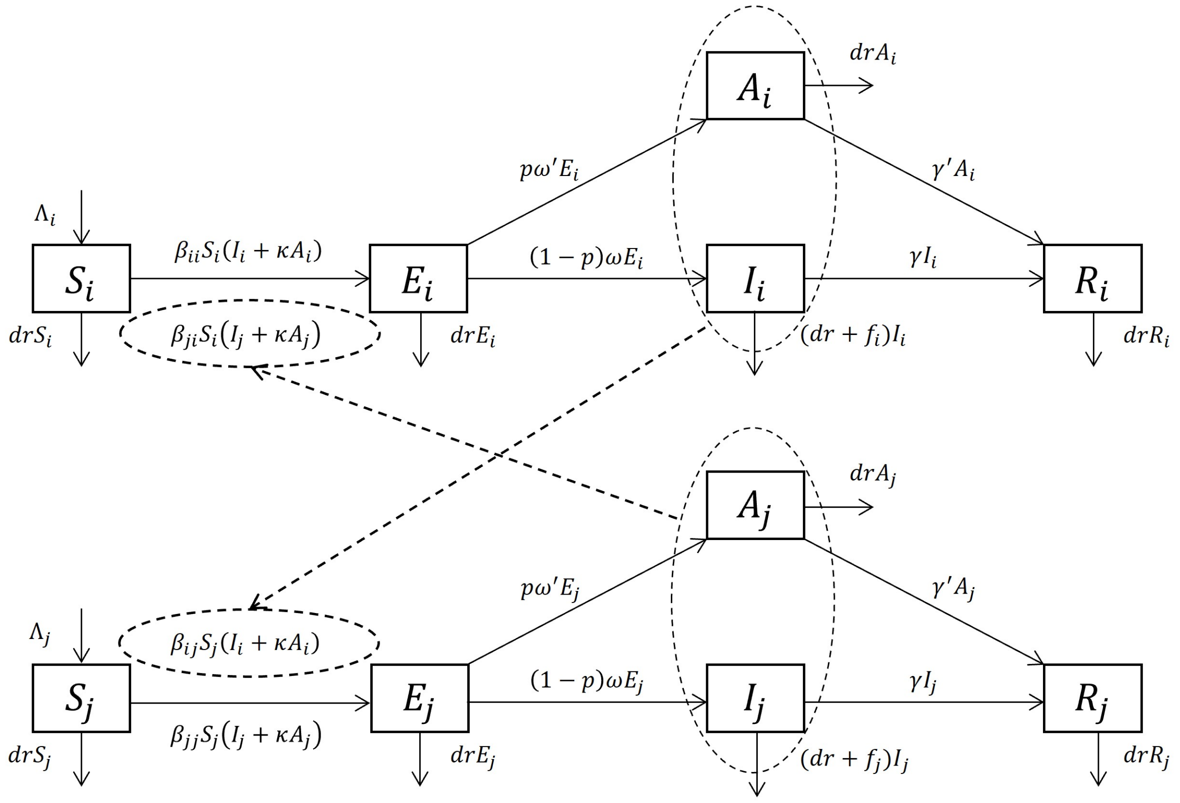

Fig. S 6: Flowchart of Multi-Group SEIAR Model. The subscript i denotes the variable or parameter is specified to the i-th group. n denotes the total number of groups. Variables S_i, E_i, I_i, A_i, R_i, $i = 1, 2, \cdots, n$ represent the susceptible, exposed, symptomatic infectious, asymptomatic infectious and recovered population in group i; $N_i = S_i + E_i + I_i + A_i + R_i$ is the population size of group i; $N = N_1 + N_2 + \cdots + N_n$ is the total population size. Parameters Λ_i is the age specific birth rate (for age-grouped model, only one Λ_i of the youngest age group is nonzero); dr_i denotes the mortality rate of group i; β_{ij} is the coefficients describing the daily transmission rate from group i to group j; κ is the relatively of transmission ability of asymptomatic cases compared with the symptomatic cases, it is assumed to be group irrelevant; f_i is the case fatality rate of group i (if group irrelevant, then f); p_i is the probability that one infected individual in group i will developed into a asymptomatic case (if group irrelevant, then p); ω_i is inverse of the average latent period of the symptomatic population in the i-th group, it is used to quantify the remove rate of compartment E_i (the inverse of incubation period is usually adopted for practice); $\omega_i^{'}$ is the inverse of the average latent period of the asymptomatic population in the i-th group; γ_i is the inverse of average infectious period for symptomatic cases; $\gamma_i^{'}$ is the inverse of average infectious period for asymptomatic.

1.7.2 Equations (multi-group SEIAR)

The corresponding ordinary differential equations are:

$$\begin{aligned}
\frac{dS_i}{dt} &= \Lambda_i - S_i \sum_{j=1}^{n} \beta_{ji} \left(I_j + \kappa A_j\right) - dr_i S_i \\
\frac{dE_i}{dt} &= S_i \sum_{j=1}^{n} \beta_{ji} \left(I_j + \kappa A_j\right) - p_i \omega_i' E_i - (1 - p_i)\, \omega_i E_i - dr_i E_i \\
\frac{dI_i}{dt} &= (1 - p_i)\, \omega_i E_i - (dr_i + f_i + \gamma_i)\, I_i \\
\frac{dA_i}{dt} &= p_i {\omega_i}' E_i - (dr_i + \gamma_i')\, A_i \\
\frac{dR_i}{dt} &= \gamma_i I_i + \gamma_i' A_i - dr_i R_i \\
i &= 1, 2, \cdots, n
\end{aligned}$$

Note that the subscript i denotes this variable or parameter is group relevant (otherwise, group irrelevant).

1.7.3 Variables and Parameters (multi-group SEIAR)

- subscript i denotes the variable or parameter is specified to the i-th group
- n denotes the total number of groups
- S_i denotes the number of susceptible population in group i
- E_i denotes the number of exposed population (i.e, infected but not infectious) population in group i
- I_i denotes the number of symptomatic infectious population in group i
- A_i denotes the number of asymptomatic infectious population in group i
- R_i denotes the number of fully immunized (i.e. impossible to be infected) population in group i
- $N_i = S_i + E_i + I_i + A_i + R_i$ is the population size of group i
- $N = N_1 + N_2 + \cdots + N_n$ is the total population size
- Λ_i denotes the age specific birth rate (for age-grouped model, only one Λ_i of the youngest age group is nonzero)
- dr_i denotes the mortality rate of group i
- β_{ij} is the coefficients describing the daily transmission rate from group i to group j
- κ is the relatively of transmission ability of asymptomatic cases compared with the symptomatic cases, it is assumed to be group irrelevant

- f_i is the case fatality rate of group i (if group irrelevant, then f)
- p_i is the probability that one infected individual in group i will developed into a asymptomatic case (if group irrelevant, then p)
- ω_i is inverse of the average latent period of the symptomatic population in the i-th group, it is used to quantify the remove rate of compartment E_i. The inverse of incubation period is usually adopted for practice
- $\omega_i^{'}$ is the inverse of the average latent period of the asymptomatic population in the i-th group
- γ_i is the inverse of average infectious period for symptomatic cases
- $\gamma_i^{'}$ is the inverse of average infectious period for asymptomatic cases

1.7.4 Derivation of R_0 (Multi-Group SEIAR Model)

Detail implementation of both the definition-based method (DBM) and the next-generation method (NGM) are included in the main text.

2 Discussion of the Normalization in Incidence

We use incidence βSN in all models, however, incidence bSI/N is also widely used. The differences of these two incidence will be discussed here.

We also implemented DBM and NGM to standard incidence rate $b * S * I/N$ or $b * S * (I + \kappa A)/N$ for SIR, SEIR, SEIAR, SIRC model, and $S_i \sum_{j=1}^{n} b_{ji} (I_j + \kappa A_j) / N_j$ for multi-group SEIAR model . It turns out that the R_0 derived by both DBM and NGM are equivalent for these two incidences, if a substitution $b = \beta * N$ is been made in the derived R0 formula. We explain this equivalence as follows.

2.1 Un-normalized Models

For the standard incidence rate $b * S * I/N$ in un-normalized models (variables S, I, ... are population size rather than a ratio in total population):

- If N is a constant, i.e. the total population does not change (E.g. compartmental models with its' birth rate and death rate are all zeros, or they are mutually balanced out) or does not change much (E.g. death rate and birth rate are nearly balanced, and in a relatively short period simulation, the size of total population is nearly constant), then these two incidence are equivalent in the sense that we can define a new parameter

$b = \beta N$ and describe the incidence either by $\beta * S * I$ or by $b * S * I/N$ without any change to the dynamical system——just a substitution of the parameter. The fitted b shall be N times of β.

- If N cannot be treated as a constant (population is converging to it's stationary size for a long-term simulation, or the fatality and the natural birth rate/death rate are not balanced), then the standard $b * S * I/N$ will be better not only because it included the variation of N, but also because it has a dimension $[time^{-1}]$, rather than $[individual^{-1} \cdot time^{-1}]$ in $\beta * S * I$

In our experiences of fitting transmission rate coefficient from incident data, b is usually around 1, while β is around 1/N, which supports the substitution obtained at beginning.

2.2 Normalized Models

Sometimes we need to normalize the dynamic system by replace all variables by the corresponding variable divided by N, such that all new variables are dimensionless. The normalization is very useful tool to improve the stability of numerical solution (especially for ill-conditioned initial value problem in ODE, which usually arise in multi-host models, where the population of two host populations may vary through magnitudes).

For the standard incidence rate b*S*I/N in normalized models: We take SIR model as an example:

- Assume that the first equation is $dS/dt = -\beta * S * I$, then letting $s = S/N$, $i = I/N$, and assume that N is a constant, we have $N * ds/dt = -\beta * (N * s) * (N * i)$, i.e. $ds/dt = -(\beta * N) * s * i$. here we may replace $\beta * N$ by b, and obtained the normalized newly infection term $b * s * i$.

- Assume that the first equation is $dS/dt = -b * S * I/N$, then letting $s = S/N$, $i = I/N$, and assume that N is a constant, we have $N * ds/dt = -\beta * (N * s) * i$, i.e. $ds/dt = -b * s * i$.

3 Source Codes(MATLAB)

All codes are now available for download from GitHub, a code hosting platform. Here is the URL: `https://github.com/1095912686/Supplementary-Codes-for-Computing-R0-of-Dynamic-Models-by-a-Definition-based-Method`

3.1 NGM for SEIAR model

```
close all; clear all; clc;

%% Define all symbolic variables and parameters involved in SEAIR model
syms kappa beta S N p omega1 omega2 gamma1 gamma2 f E A I d_r;  % define symbolic variables
```

```
%% Define infected variables
variables = [E; A; I]; % all infected cells
dim = numel(variables);
F = sym(zeros(dim,1));  % initialize the vector of newly infections
V = sym(zeros(dim,1));  % initialize the vector of transitions (exluding newly infections)

% Define vector F and V
% Note that the original ODEs can be written as  dx/dt = F - V
F(1) = beta*S*(I+kappa*A);
V(1) = p*omega2*E + (1-p)*omega1*E + d_r*E;
V(2) = -p*omega2*E + gamma2*A + d_r*A;
V(3) = -(1-p)*omega1*E + (f + gamma1)*I + d_r*I;



%% construct the next generation matrix and compute its leading eigenvalue.
% compute the jacobian matrix
JF = sym(zeros(dim));
JV = sym(zeros(dim));
for i = 1:dim
    for j = 1:dim
        JF(i,j) = diff(F(i),variables(j));
        JV(i,j) = diff(V(i),variables(j));
    end
end

% construct the next-generation matrix Mat = F*V^(-1)
Mat = JF*inv(JV)

% all eigenvalues of the Next-Generation matrix
eigenvalues = eig(Mat)

% maximum real part of eigenvalues
Reff = eigenvalues(end)

% R0 is obtained at the disease free equalibrium (N, 0, 0, 0, 0, 0, 0)
R0 = subs(Reff,S,N)


pretty(Reff)
```

```
latex(Reff)
```

3.2 NGM for grouped SEIAR model

```
close all; clear; clc;
format long;

%% Define all symbolic variables and parameters involved in (SEIAR)_i^n
n = 4;  % number of groups
syms S1 S2 E1 E2 I1 I2 A1 A2 R1 R2;
syms S3 E3 I3 A3 R3;
syms S4 E4 I4 A4 R4;
syms beta11 beta21 beta12 beta22;
syms beta13 beta23 beta33 beta31 beta32;
syms beta14 beta24 beta34 beta44 beta41 beta42 beta43;
syms omega1 omega2 gamma1 gamma2 p kappa f1 f2 f3 f4;


%% Define infected variables
variables = [E1,A1,I1,E2,A2,I2,E3,A3,I3,E4,A4,I4]; % all infected cells
dim = numel(variables);   % dimension, number of infected variables
F = sym(zeros(dim,1));  % initialize the vector of newly infections
V = sym(zeros(dim,1));  % initialize the vector of transitions (exluding newly infections)

% Define vector F and V
% Note that the original ODEs can be written as  dx/dt = F - V
F(1) = beta11*S1*(I1+kappa*A1) + beta21*S1*(I2+kappa*A2) + beta31*S1*(I3+kappa*A3) + ...
    beta41*S1*(I4+kappa*A4);
V(1) = p*omega2*E1 + (1-p)*omega1*E1;
V(2) = -p*omega2*E1 + gamma2*A1;
V(3) = -(1-p)*omega1*E1 + (f1+gamma1)*I1;

F(4) = beta22*S2*(I2+kappa*A2) + beta12*S2*(I1+kappa*A1) + beta32*S2*(I3+kappa*A3) + ...
    beta42*S2*(I4+kappa*A4);
V(4) = p*omega2*E2 + (1-p)*omega1*E2;
V(5) = -p*omega2*E2 + gamma2*A2;
V(6) = -(1-p)*omega1*E2 + (f2+gamma1)*I2;

```

```
F(7) = beta33*S3*(I3+kappa*A3) + beta13*S3*(I1+kappa*A1) + beta23*S3*(I2+kappa*A2) + ...
    beta43*S3*(I4+kappa*A4);
V(7) = p*omega2*E3 + (1-p)*omega1*E3;
V(8) = -p*omega2*E3 + gamma2*A3;
V(9) = -(1-p)*omega1*E3 + (f3+gamma1)*I3;

F(10) = beta44*S4*(I4+kappa*A4) + beta14*S4*(I1+kappa*A1) + beta24*S4*(I2+kappa*A2) + ...
    beta34*S4*(I3+kappa*A3);
V(10) = p*omega2*E4 + (1-p)*omega1*E4;
V(11) = -p*omega2*E4 + gamma2*A4;
V(12) = -(1-p)*omega1*E4 + (f4+gamma1)*I4;

%% construct the next generation matrix
% compute the jacobian matrices JF, JV via differentiating F, V on variables
JF = sym(zeros(dim));
JV = sym(zeros(dim));
for i = 1:dim
    for j = 1:dim
        JF(i,j) = diff(F(i),variables(j));
        JV(i,j) = diff(V(i),variables(j));
    end
end

% construct the next-generation matrix Mat = F*V^(-1)
invJV = inv(JV)
Mat = JF * invJV


%% Compute the leading eigenvalues of sub-matrices in the next-generation matrix
% assume that {omega1 omega2 gamma1 gamma2 p kappa} are group irrelevent, and f1 = f2 = f3 = f4
C = sym(zeros(3));
C(1,1) = (kappa*omega2*p)/(gamma2*(omega1 - omega1*p + omega2*p)) - (omega1*(p - 1))/((f1 + ...
    gamma1)*(omega1 - omega1*p + omega2*p));
C(1,2) = kappa/gamma2;
C(1,3) = 1/(gamma1+f1);

% by defining the following symbolic Beta matrix with B(i,j) = betaji * Si,
% and denote B(i,j) as betaij)
for i = 1:n
    for j = 1:n
        B(i,j) = eval(['sym(beta',num2str(j),num2str(i),')']);
```

```
    end
end

% then the next-generation matrix can be formularized as: Mat = kron(B, C)
% thus the interactive Rij given by eigenvalues of sub-matrices is
% B(i,j)*max(eig(C)), which leads to formula (6) in paper.
%

% since matrix C has only one non-zeros row, i.e. the first row, then C has
% only one non-zero eigenvalue: C(1,1)
%

% once values of all parameters are obtained, one can use formula (8) in paper:
% R0 = max(eig(eval(B))) * max(eig(eval(C)))
% (note that for R0, we shall set S1 = N1,...,Sn = Nn in substitution)
%


%% Numerical Experiment proves the derivation is correct
% The derivation may be complicate to check from one cell to another,
% therefore, a numerical experiment is designed based on random-generated
% parameters. If the derivation is correct, then the result from our derivation
% should identical to the result of implicit form.
%

% random generated Beta matrix and parameters
for i = 1:n
    for j = 1:n
        eval(['beta',num2str(j),num2str(i),'= rand(1);']);
    end
end

omega1 = rand(1);
omega2 = rand(1);
gamma1 = rand(1);
gamma2 = rand(1);
p = rand(1);
kappa = rand(1);
f1 = rand(1);
f2 = f1;
f3 = f1;
```

```
f4 = f1;
S1 = 1;
S2 = 1;
S3 = 1;
S4 = 1;



% substitute parameteres (Note that the population size is included in
% betaij in Line 66-67. )
eMat = eval(Mat);
eB = eval(B);
eC = eval(C);
eJF = eval(JF);
eJV = eval(JV);
eJV1 = eJV(1:3,1:3);


% differences between the implicit R0 via NGM and the derived explicit R0 via NGM
err = eMat - kron(eB,eC);
normErr = norm(err)



% all blocks of Next-Generation matrix are identtial
for i = 1:n
    for j = 1:n
        eval(['err_eMat',num2str(i),num2str(j),' = norm(eJF( 3*i-2:3*i , 3*j-2:3*j )*inv(eJV1) - ...
            eMat( 3*i-2:3*i , 3*j-2:3*j ))']);
    end
end


% R0 are identical
R1 = max(eig(eMat))
R2 = max(eval(eig(B)))*max(eval(eig(C)))


% Notes: the Matlab function latex() is a useful tool that transform the
% symbolic expression into latex codes, and combine with Mathpix, it will
% be convienient to paste the complecated formula into a Microsoft Word
```

```
% document.
```

3.3 NGM for SIRC model

```
close all; clear; clc;
format long;

%% Define all symbolic variables and parameters involved in the SIRC model
syms S I R C N;
syms Gamma a p gamma mu beta;

%% Define infected variables
variables = [I C]; % all infected cells
dim = numel(variables);
F = sym(zeros(dim,1));  % initialize the vector of newly infections
V = sym(zeros(dim,1));  % initialize the vector of transitions (exluding newly infections)

% Define vector F and V
% Note that the original ODEs can be written as  dx/dt = F - V
F(1) = beta*S*(I+C);
V(1) = (a + gamma)*I;
V(2) = -p*gamma*I + (mu + a)*C;




%% construct the next generation matrix and compute its leading eigenvalue.
% compute the jacobian matrices JF, JV via differentiating F, V on variables
JF = sym(zeros(dim));
JV = sym(zeros(dim));
for i = 1:dim
    for j = 1:dim
        JF(i,j) = diff(F(i),variables(j));
        JV(i,j) = diff(V(i),variables(j));
    end
end

% construct the next-generation matrix Mat = F*V^(-1)
invJV = inv(JV)
```

```
Mat = JF*invJV

% all eigenvalues of the Next-Generation matrix
eigenvalues = eig(Mat)

% maximum real part of eigenvalues
Reff = eigenvalues(end)

% R0 is obtained at the disease free equalibrium (N, 0, 0, 0, 0, 0, 0)
R0 = subs(Reff,S,N)
```

3.4 NGM for SEIARW model

```
close all; clear all; clc;

%% Define all symbolic variables and parameters involved in SEIARW model
syms beta beta_w p omega1 omega2 mu1 mu2 gamma1 gamma2 epsilon k dr;
syms S E I A R W N;

%% Define infected variables
variables = [E, I, A, W]; % all infected cells
dim = numel(variables);
F = sym(zeros(dim,1));  % initialize the vector of newly infections
V = sym(zeros(dim,1));  % initialize the vector of transitions (exluding newly infections)

% Define vector F and V
% Note that the original ODEs can be written as  dx/dt = F - V
F(1) = beta*S*(I+k*A) + beta_w*S*W;
V(1) = (1-p)*omega1*E + p*omega2*E + dr*E;
V(2) = -(1-p)*omega1*E + gamma1*I + dr*I;
V(3) = -p*omega2*E + gamma2*A + dr*A;
V(4) = -mu1*I - mu2*A + epsilon*W;



%% construct the next generation matrix and compute its leading eigenvalue.
% compute the jacobian matrix
JF = sym(zeros(dim));
JV = sym(zeros(dim));
```

```
for i = 1:dim
    for j = 1:dim
        JF(i,j) = diff(F(i),variables(j));
        JV(i,j) = diff(V(i),variables(j));
    end
end
JF
JV

%% the next-generation matrix F*Vˆ(-1)
% the matrix is in fact the reproduction matrix for interactive
% transmissibility
Mat = JF*inv(JV)

% all eigenvalues of the Next-Generation matrix
eigenvalues = eig(Mat)

% maximum real part of eigenvalues
Reff = eigenvalues(end)

% R0 is obtained at the disease free equalibrium (N, 0, 0, 0, 0, 0, 0)
R0 = subs(Reff,S,N)

% latex code
latex(R0)
```

3.5 Data Validation for DBM and NGM for R_0 for entire population

```
%% Evaluation R_ij and R_0 for Covid-19 in Hunan province via DBM and NGM
clear; clc; close all;

omega1 = 0.1429;
omega2 = 0.1429;
gamma1 = 0.2;
gamma2 = 0.2;
f = 3.552e-3;
p = 0.2015;
kappa = 1;

```

```
% matrices of beta_ij
Beta_med = [1.70e-13   1.12e-12    4.10e-10    1.66e-11;...
            7.16e-12   1.24e-9     1.21e-16    1.45e-10;...
            6.63e-11   1.46e-11    1.64e-19    7.71e-9; ...
            7.20e-14   3.61e-10    3.07e-9     3.66e-17    ]; % the entry-wise median of Beta matrix

Beta_1 = [3.62e-16  1.43e-11    5.15e-13    3.69e-14;...
          3.06e-13  8.97E-09    2.58e-15    1.41e-11;...
          8.38e-12  1.51e-17    5.34e-20    5.43e-9; ...
          3.46E-11  5.88E-11    9.81E-08    2.83E-17       ];



% population size of Hunan and Jilin Province
N_hunan = [13618898   26623844    20035661    8709900];
N_jilin = [3291955, 10827458, 9525131, 3395414];
P = N_hunan / sum(N_hunan);

Beta_med = Beta_med.*N_hunan;
Beta_1 = Beta_1 .* N_hunan;


c = kappa*omega2*p/(gamma2*(omega1 - omega1*p + omega2*p)) + omega1*(1 - p)/((f + ...
    gamma1)*(omega1 - omega1*p + omega2*p));

% matrix of R_ij
Rmataver = Beta_med*c
Rmat1 = Beta_1*c



R0_NGM_1 = max(eig(Beta_1)) * c
R0_DEF_1 = sum(P'.*sum(Rmat1,2))


%% For all time segments from 1 to 4
% read Betas
betaTable = readtable('Betas.xlsx');
betaTable(:,1) = [];
names = betaTable.Properties.VariableNames;
```

```

% population
N_hunan = [13618898   26623844    20035661    8709900];
P_hunan = N_hunan / sum(N_hunan);

% add max beta
beta_max = max(betaTable{:,:});
betaTable = [betaTable; mat2cell(beta_max,1,ones(1,16))];


%%% compute R_ij matrix and R0 on Beta matrices for time segments from 1 to 4
% from time segment 1 to 4
h1 = figure;
for i = 1:4
    % extract Beta matrix B from table
    B = zeros(4);
    for k = 1:16
        ii = eval(names{k}(2));
        jj = eval(names{k}(3));
        B(ii,jj) = betaTable{i,k};
    end

    %%% compute the R_ij matrix and R0 using DBM and NGM
    % compute the constant coefficient c (which is identical in DBM and NGM, due to the adoption ...
        of the group-irrelevant parameters )
    c = kappa*omega2*p/(gamma2*(omega1 - omega1*p + omega2*p)) + omega1*(1 - p)/((f + ...
        gamma1)*(omega1 - omega1*p + omega2*p));

    % R_ij matrix for time segment i
    Rmat = B .* N_hunan * c;

    % R0 for time segment i
    fprintf('\n \n R0 for time segment %d: \n', i);
    R0_DEF = sum(P_hunan'.*sum(Rmat,2))    % definition-based method
    R0_NGM = max(eig(B.*N_hunan)) * c      % next-generation method


    %%% visualizing the R_ij matrix by heatmaps
    subplot(2,2,i);     % plot the heatmap for time segment i
    format shorte;
    xvalue = {'0 to 14','15 to 44','45 to 64', '\geq 65'};
```

```
    yvalue = {'0 to 14','15 to 44','45 to 64', '\geq 65'};
    h_DEF = heatmap(xvalue,yvalue,Rmat);
    cellDate = {'Jan. 5 2021', 'Jan. 25 2021', 'Jan. 31 2021', 'Feb. 5 2021', 'Feb. 19 2021'};

%   h_DEF.Title = ['R_{ij} matrix, time segment ',num2str(i),' from ',cellDate{i},' to ...
    ',cellDate{i+1}];
    h_DEF.Title = ['R_{ij} Matrix of Time Segment ',num2str(i)];
    h_DEF.XLabel = 'to age group j';         % to the entirely susceptiable age group j
    h_DEF.YLabel = 'from age group i';       % from one infected individual in age group i

    h_DEF.FontName = 'Times New Roman';
    h_DEF.CellLabelFormat = '%.4e ';
%     c = colorbar;
%     c.Label.String = 'interactive R_{ij} between different groups';

    % save record
    R0_DEFs(i,1) = R0_DEF;
    R0_NGMs(i,1) = R0_NGM;
    eval(['B',num2str(i),'=','B;']);
    eval(['Rmat',num2str(i),'=','Rmat;']);
    eval(['R0_DEF',num2str(i),'=','R0_DEF;']);
    eval(['R0_NGM',num2str(i),'=','R0_NGM;']);
end

% heat map of increasement of Rij matrices (from time segment 1 to 4)
h2 = figure;
for i = 2:4
    subplot(3,1,i-1);
    format shorte;
    xvalue = {'0 to 14','15 to 44','45 to 64', '\geq 65'};
    yvalue = {'0 to 14','15 to 44','45 to 64', '\geq 65'};
    h_DEF = heatmap(xvalue,yvalue,eval([' Rmat',num2str(i),' - Rmat',num2str(i-1)]));
    h_DEF.Title = ['The matrix of increasement of R_{ij}, time segment ',num2str(i-1),' to ...
        sement ',num2str(i)];
    h_DEF.XLabel = 'In the entirely susceptiable age group j';
    h_DEF.YLabel = 'One infected individual in age group i';
    h_DEF.CellLabelFormat = '%.4e ';
end


% print(h1,'-dpdf','h1.pdf','-r300');
```

```
% print(h2,'-dpdf','h2.pdf','-r300');
```
